# Global burden, trends, and projections to 2050 of neuroblastoma and other peripheral nervous cell tumors: a systematic analysis of the global burden of disease study from 1990 to 2021

**DOI:** 10.3389/fped.2025.1604053

**Published:** 2025-09-03

**Authors:** Hang Wu, Jingjing Li, Yue Yang, Xiaoqi Xuan, Jinlong Yang

**Affiliations:** ^1^ Department of Pediatric Urology, Affiliated Children’s Hospital of Jiangnan University (Wuxi Children’s Hospital), Wuxi, Jiangsu, China; ^2^ Department of Infectious Diseases, Affiliated Children’s Hospital of Jiangnan University (Wuxi Children’s Hospital), Wuxi, Jiangsu, China

**Keywords:** neuroblastoma, peripheral nervous cell tumors, disability-adjusted life years (DALYs), global burden of disease, predictive modeling, epidemiological trends

## Abstract

**Background:**

Neuroblastoma and other Peripheral Nervous Cell Tumors (NPNTs) contribute substantially to global pediatric cancer morbidity and mortality, particularly among children under five. This study provides a comprehensive analysis of the global burden of NPNTs, examining long-term trends from 1990 to 2021 and projecting future patterns through 2050, based on data from the Global Burden of Disease (GBD) Study 2021.

**Methods:**

We analyzed mortality and disability-adjusted life years (DALYs) from 1990 to 2021 using GBD data. Trends were assessed via age-standardized rates (ASRs) and estimated annual percentage change (EAPC). Predictive models (Exponential Smoothing and ARIMA) projected future burden through 2050. Analyses were stratified by age, sex, and socio-demographic index (SDI) regions.

**Results:**

In 2021, NPNTs resulted in 5,194 deaths (95% UI: 4,295–5,932) and 285,479 DALYs (95% UI: 227,709–341,110) globally. Children under five years accounted for 1,355 deaths (26.1% of total) and 126,215 DALYs (44.2% of total), with males exhibiting higher mortality rates (ASR: 0.08 vs. 0.06 per 100,000 in females). Middle-SDI regions experienced the highest number of deaths (1,503) and DALYs (79,412), while high-SDI regions had the highest age-standardized death rate (0.09 per 100,000) and DALYs rate (5.25 per 100,000). From 1990 to 2021, population growth drove 90.2% of DALYs increases, while aging offset 13.0% of the rise. Projections diverged: ES models predicted stable trends, whereas ARIMA forecasted a 22.3% increase in male DALYs by 2050 (from 165,574 to 226,533).

**Conclusions:**

The escalating burden of NPNTs is strongly tied to demographic expansion and inequitable healthcare access. Prioritizing early diagnosis in high-burden regions (e.g., middle/low-SDI settings) and addressing sex-specific disparities are imperative. These findings call for data-driven policies to mitigate future disease burden through targeted resource allocation and surveillance strengthening.

## Introduction

Neuroblastoma and other Peripheral Nervous Cell Tumors (NPNTs) are a group of neoplasms originating from the peripheral nervous system, predominantly affecting pediatric populations. These tumors encompass neuroblastoma, ganglioneuroma, and ganglioneuroblastoma, spanning a spectrum from highly aggressive malignancies to relatively benign subtypes (1). A common characteristic of these tumors is their derivation from the sympathetic nervous chain, which contributes to their complex pathological and biological features. The clinical presentation of NPNTs is highly variable, often resulting in delayed diagnoses that negatively impact treatment outcomes and long-term prognosis (2).

As one of the most common solid tumors in children, NPNTs account for a significant proportion of cancer-related deaths, particularly among children under five years old. The global burden of NPNTs varies substantially across regions and socioeconomic settings. High-income countries have achieved notable improvements in survival rates due to advanced diagnostic tools and access to specialized therapies, with survival exceeding 70% in many cases (3). Conversely, low- and middle-income countries face significant challenges, including delayed diagnoses, limited treatment options, and systemic healthcare inequities. These disparities are especially pronounced in Asia and Africa, where the burden of childhood cancers is disproportionately high compared to the availability of healthcare resources (4).

Gender and age disparities further characterize the epidemiology of NPNTs. Males consistently exhibit higher disease burdens than females, a pattern that may be attributable to genetic and hormonal factors as well as differences in healthcare access and behaviors. Moreover, NPNTs are predominantly a disease of young children, with the majority of diagnoses occurring before the age of five. This emphasizes the critical importance of early screening programs and timely interventions to improve outcomes in this vulnerable population (5, 6).

While previous studies have documented survival disparities linked to socioeconomic factors and healthcare access, critical gaps persist in understanding the global drivers of these disparities and their long-term trajectories (7). Despite advances in treatment, no study to date has systematically quantified the contributions of population dynamics vs. epidemiological changes to NPNTs burden over time—a gap that limits evidence-based policy formulation (6). While significant progress has been made in the treatment of childhood cancers over recent decades, there is limited research quantifying the temporal trends in NPNTs burden or predicting its future trajectory. These gaps hinder the development of effective global health policies and resource allocation strategies.

Given the critical role of Neuroblastoma and other Peripheral Nervous Cell Tumors (NPNTs) in contributing to the global pediatric cancer burden, a more comprehensive understanding of their distribution, trends, and drivers is essential. This study addresses these gaps by conducting a systematic analysis of NPNTs, focusing on age, gender, regional, and national variations in disease burden. It also examines temporal trends and quantifies the relative contributions of key drivers, such as population growth and epidemiological improvements, to past and future changes. By providing forward-looking projections, this research aims to inform global health policies and support targeted interventions for reducing the burden of NPNTs worldwide.

## Methods

### Overview and methodological details

This study evaluated the mortality and disability-adjusted life years (DALYs) of Neuroblastoma and other Peripheral Nervous Cell Tumors (NPNTs) to assess the global burden of disease from 1990 to 2021. Data were sourced from the Global Burden of Disease (GBD) 2021 database, which provides comprehensive epidemiological estimates across regions and countries. The analysis included stratifications by age groups, gender, and Socio-Demographic Index (SDI) categories. This study strictly adheres to the IHME Terms of Use for non-commercial applications. All GBD data were accessed and analyzed in accordance with the IHME's free-of-charge, non-commercial user agreement, ensuring compliance with restrictions on redistribution and commercial use.

DALYs, a key indicator of disease burden, were calculated as the sum of Years of Life Lost (YLLs) due to premature mortality and Years Lived with Disability (YLDs), using the formula: DALYs=YLLs+YLDs. YLLs were computed by multiplying the number of deaths (N) by the standard life expectancy at the age of death (L): YLLs=N×L. YLDs were estimated by multiplying the prevalence (P) of the condition by a corresponding disability weight (DW): YLDs=P×DW. Disability weights range from 0 (perfect health) to 1 (death) and are derived from population-based surveys and expert consultations conducted by the Global Burden of Disease study. These formulas enable a comprehensive quantification of both fatal and non-fatal health loss. Population estimates required for calculating age-standardized rates (ASRs) were sourced from the GBD database, ensuring consistency in demographic data. Stratifications included five age groups (<1 year, 1–4 years, 5–9 years, 10–14 years, and 15–19 years) to capture the burden across developmental stages.

The data for this analysis were obtained from the GBD Results tool. The citation for the data used in this study is as follows:

Global Burden of Disease Collaborative Network.

Global Burden of Disease Study 2021 (GBD 2021) Results.

Seattle, United States: Institute for Health Metrics and Evaluation (IHME), 2022.

Available from: https://vizhub.healthdata.org/gbd-results/.

## Statistical analysis

Temporal trends in age-standardized rates (ASRs) for NPNTs-related mortality and DALYs were assessed using the Estimated Annual Percentage Change (EAPC) (8). EAPC provides the average annual rate of change over a specified period and is calculated using the following formula:

$$\mathrm{EAPC}=(e^{\beta }-1)\times 100$$

where β is the slope of the natural logarithm of the ASR over time, derived from the linear regression model:

ln(ASR)=β×Year+α

Here, α is the intercept. A positive EAPC indicates an increasing trend, while a negative EAPC suggests a decline. This measure captures long-term variations in NPNTs burden from 1990 to 2021.

To explore global and regional disparities, data were stratified by SDI and geographic regions as defined by the GBD framework (9). Heatmaps and visualizations were generated to illustrate spatial variations in mortality and DALYs, highlighting regions with high and low disease burden. We applied a decomposition method that disaggregates the total change into three components: population growth, population aging, and epidemiological changes. This method has been widely used in GBD-based studies to isolate the influence of demographic and epidemiological dynamics (10). The total difference in DALYs (or deaths) between 1990 and 2021 (ΔTotal) was decomposed as:

$$\Delta_{\mathrm{Total}}=\Delta_{\mathrm{Population}}+\Delta_{\mathrm{Aging}}+\Delta_{\mathrm{Epi}}$$

Where ΔPopulation represents the change due to overall population growth, holding age structure and age-specific rates constant by applying the 1990 age structure and age-specific rates to the 2021 total population; ΔAging captures the effect of shifts in age distribution by applying the 2021 age structure and 1990 age-specific rates to the 2021 population; and ΔEpi is the residual component reflecting changes in age-specific DALY or death rates, such as improvements or deterioration in disease control, diagnostics, or treatment. As a worked example, if the global number of DALYs in 1990 was 100,000 and increased to 150,000 by 2021, the decomposition might show the contribution of population growth as 30,000, aging as −5,000, and epidemiological changes as 25,000. This suggests that demographic factors including growth and aging contributed a net increase of 25,000, while changes in health-related conditions accounted for the remaining 25,000.

Future trends in NPNTs burden were predicted using two different time-series models. The Exponential Smoothing (ES) model was selected for its ability to capture trend and seasonal components in time-series data, particularly suitable for stable epidemiological trends observed in high-SDI regions. In contrast, the ARIMA model was chosen to account for temporal dependencies and non-stationary trends, offering robustness for regions with volatile healthcare infrastructure. This dual-model approach mitigates the limitations of single-model projections by providing complementary scenarios. The ARIMA model was specified as ARIMA(1,1,0) based on the inspection of autocorrelation and partial autocorrelation plots. Stationarity was assessed using the Augmented Dickey–Fuller test. Model selection was guided by minimizing the Akaike Information Criterion (AIC) and Bayesian Information Criterion (BIC), and model performance was evaluated using the root mean square error (RMSE). Out-of-sample validation and residual diagnostics were not conducted, as the full dataset from 1990 to 2021 was used to optimize model stability and ensure maximum use of available data for long-term projections.

All statistical analyses in this study were conducted using R language (version 4.3.3). Descriptive statistics for mortality and DALYs were summarized as means with 95% confidence intervals (CI). Observed trends were evaluated using appropriate statistical methods, with a *p*-value < 0.05 considered statistically significant. Predictive modeling results offered actionable insights into the future burden of NPNTs.

## Results

### The disease burden attributable to neuroblastoma and other peripheral nervous cell tumors (NPNTs) in 2021

In 2021, neuroblastoma and other peripheral nervous cell tumors (NPNTs) caused 5,194 deaths globally (95% UI: 4,295–5,932), corresponding to an age-standardized death rate (ASR) of 0.07 per 100,000 (95% UI: 0.06–0.08), and 285,479 disability-adjusted life years (DALYs) (95% UI: 227,709–341,110), with an age-standardized DALYs rate of 3.95 per 100,000 (95% UI: 3.11–4.77) (Tables 1, 2).

**Table 1:** The number of deaths cases and the age-standardized deaths rate attributable to neuroblastoma and other peripheral neuroblastoma in 1990 and 2021, and its trends from 1990 to 2021 globally.

Characteristics	1990		2021		1990–2021
	Number of deaths cases(95% UI)	The age-standardized deaths rate/100,000 (95% UI)	Number of deaths cases(95% UI)	The age-standardized deaths rate/100,000 (95% UI)	EAPC(95% CI)
Global	2,675 (2,298–3,138)	0.05 (0.04–0.06)	5,194 (4,295–5,932)	0.07 (0.06–0.08)	0.93 (0.85–1.01)
Sex					
Both	2,675 (2,298–3,138)	0.05 (0.04–0.06)	5,194 (4,295–5,932)	0.07 (0.06–0.08)	0.93 (0.84–1.02)
Female	1,223 (920–1,547)	0.05 (0.04–0.06)	2,213 (1,741–2,628)	0.06 (0.04–0.07)	0.71 (0.62–0.81)
Male	1,452 (1,263–1,628)	0.06 (0.05–0.06)	2,981 (2,477–3,469)	0.08 (0.07–0.09)	1.12 (1.03–1.21)
Age					
<5 years	1,189 (969–1,441)	0.19 (0.16–0.23)	1,355 (929–1,830)	0.21 (0.14–0.28)	0.42 (0.3–0.54)
10–14 years	109 (89–134)	0.02 (0.02–0.03)	181 (142–208)	0.03 (0.02–0.03)	0.94 (0.86–1.03)
15–19 years	69 (57–86)	0.01 (0.01–0.02)	118 (98–139)	0.02 (0.02–0.02)	0.97 (0.82–1.12)
5–9 years	345 (303–401)	0.06 (0.05–0.07)	441 (347–519)	0.06 (0.05–0.08)	0.42 (0.31–0.53)
SDI regions					
High-middle SDI	614 (510–732)	0.06 (0.05–0.07)	1,168 (964–1,318)	0.08 (0.07–0.09)	0.87 (0.77–0.97)
High SDI	774 (732–808)	0.1 (0.09–0.1)	1,048 (956–1,124)	0.09 (0.08–0.1)	−0.39 (−0.65–0.13)
Low-middle SDI	443 (342–581)	0.03 (0.02–0.04)	953 (726–1,204)	0.05 (0.04–0.07)	1.92 (1.79–2.05)
Low SDI	199 (134–317)	0.03 (0.02–0.04)	517 (295–813)	0.04 (0.02–0.06)	1.38 (0.98–1.77)
Middle SDI	642 (530–737)	0.04 (0.03–0.04)	1,503 (1,238–1,710)	0.06 (0.05–0.07)	1.83 (1.68–1.98)
GBD regions					
Advanced health system	1,160 (1,060–1,254)	0.09 (0.09–0.1)	1,499 (1,361–1,607)	0.09 (0.08–0.1)	−0.07 (−0.24–0.1)
Africa	291 (212–425)	0.03 (0.02–0.05)	761 (440–1,132)	0.05 (0.03–0.07)	1.19 (0.86–1.52)
African region	251 (168–374)	0.03 (0.02–0.05)	691 (375–1,052)	0.05 (0.03–0.08)	1.26 (0.88–1.64)
America	581 (548–611)	0.08 (0.08–0.09)	882 (799–966)	0.09 (0.08–0.1)	0.27 (0.03–0.51)
Andean Latin America	25 (19–31)	0.06 (0.05–0.07)	46 (36–58)	0.07 (0.06–0.09)	0.72 (0.61–0.83)
Asia	1,092 (866–1,358)	0.03 (0.03–0.04)	2,651 (2,140–3,112)	0.06 (0.05–0.07)	1.88 (1.71–2.06)
Australasia	19 (17–21)	0.1 (0.09–0.11)	31 (26–37)	0.09 (0.08–0.11)	−0.07 (−0.27–0.13)
Basic health system	836 (726–981)	0.04 (0.03–0.04)	2,083 (1,718–2,364)	0.07 (0.05–0.08)	2.04 (1.88–2.2)
Caribbean	22 (18–28)	0.06 (0.05–0.07)	42 (34–54)	0.09 (0.07–0.12)	1.5 (1.23–1.78)
Central Africa	12 (8–17)	0.01 (0.01–0.02)	18 (12–31)	0.01 (0.01–0.02)	−0.67 (−1.02–0.33)
Central Asia	15 (11–19)	0.02 (0.02–0.03)	47 (38–57)	0.05 (0.04–0.06)	3.38 (3.08–3.69)
Central Europe	84 (75–95)	0.07 (0.06–0.08)	115 (102–131)	0.08 (0.07–0.09)	0.58 (0.21–0.94)
Central Latin America	101 (93–109)	0.06 (0.05–0.06)	176 (151–203)	0.07 (0.06–0.08)	0.52 (0.13–0.9)
Central Sub-Saharan Africa	9 (5–15)	0.01 (0.01–0.02)	19 (14–28)	0.02 (0.01–0.02)	0.14 (−0.14–0.43)
Commonwealth high income	135 (129–141)	0.13 (0.13–0.14)	149 (137–160)	0.1 (0.09–0.1)	−0.7 (−0.88–0.52)
Commonwealth low income	112 (80–167)	0.03 (0.02–0.05)	226 (123–350)	0.05 (0.03–0.08)	1.14 (0.65–1.63)
Commonwealth middle income	499 (307–686)	0.04 (0.02–0.05)	1,263 (904–1,635)	0.06 (0.05–0.08)	1.68 (1.45–1.91)
East Asia	333 (267–422)	0.03 (0.03–0.04)	1,100 (821–1,328)	0.07 (0.05–0.08)	3.26 (2.94–3.57)
East Asia & Pacific—WB	627 (530–745)	0.04 (0.03–0.04)	1,630 (1,291–1,885)	0.06 (0.05–0.07)	2.14 (1.95–2.33)
Eastern Africa	78 (52–142)	0.03 (0.02–0.05)	201 (107–332)	0.04 (0.02–0.07)	1.28 (0.72–1.83)
Eastern Europe	182 (128–244)	0.08 (0.06–0.11)	204 (176–232)	0.08 (0.07–0.09)	−0.64 (−0.97–0.31)
Eastern Mediterranean Region	153 (110–225)	0.03 (0.02–0.04)	455 (325–651)	0.06 (0.04–0.08)	2.29 (2.02–2.56)
Eastern Sub-Saharan Africa	121 (84–201)	0.04 (0.03–0.06)	280 (144–452)	0.05 (0.03–0.08)	0.94 (0.34–1.55)
Europe	706 (631–787)	0.09 (0.08–0.1)	893 (801–975)	0.09 (0.08–0.1)	0.09 (−0.08–0.25)
Europe & Central Asia—WB	716 (638–799)	0.09 (0.08–0.1)	924 (830–1,010)	0.09 (0.08–0.1)	0.09 (−0.07–0.26)
European Region	722 (645–806)	0.09 (0.08–0.1)	933 (838–1,021)	0.09 (0.08–0.1)	0.09 (−0.08–0.25)
High-income Asia Pacific	136 (125–148)	0.1 (0.09–0.11)	178 (163–192)	0.1 (0.09–0.11)	−0.1 (−0.4–0.21)
High-income North America	277 (257–294)	0.11 (0.1–0.11)	346 (314–372)	0.09 (0.08–0.1)	−0.25 (−0.44–0.07)
Latin America & Caribbean—WB	305 (285–328)	0.07 (0.06–0.07)	538 (477–611)	0.09 (0.08–0.1)	0.7 (0.41–1)
Limited health system	645 (438–893)	0.03 (0.02–0.04)	1,550 (1,084–2,043)	0.05 (0.04–0.07)	1.5 (1.22–1.78)
Middle East & North Africa—WB	79 (61–104)	0.03 (0.02–0.03)	153 (119–197)	0.04 (0.03–0.05)	0.84 (0.72–0.96)
Minimal health system	31 (21–50)	0.01 (0.01–0.02)	57 (31–104)	0.01 (0.01–0.03)	−0.29 (−0.83–0.24)
North Africa and Middle East	122 (90–170)	0.03 (0.02–0.04)	239 (188–299)	0.04 (0.03–0.05)	0.96 (0.83–1.09)
North America	277 (257–294)	0.11 (0.1–0.11)	346 (314–372)	0.09 (0.08–0.1)	−0.25 (−0.44–0.07)
Northern Africa	52 (39–72)	0.04 (0.03–0.05)	82 (60–119)	0.04 (0.03–0.06)	0.19 (0.02–0.35)
Oceania	0 (0–1)	0.01 (0.01–0.02)	1 (1–1)	0.01 (0.01–0.01)	−1.01 (−1.44–0.57)
Region of the Americas	581 (548–611)	0.08 (0.08–0.09)	882 (799–966)	0.09 (0.08–0.1)	0.27 (0.03–0.51)
South-East Asia Region	415 (293–552)	0.03 (0.02–0.04)	778 (625–928)	0.04 (0.03–0.05)	0.77 (0.58–0.96)
South Asia	420 (284–578)	0.03 (0.02–0.04)	901 (679–1,168)	0.05 (0.04–0.07)	1.43 (1.18–1.68)
South Asia—WB	428 (291–588)	0.03 (0.02–0.04)	919 (692–1,191)	0.05 (0.04–0.07)	1.4 (1.16–1.63)
Southeast Asia	147 (114–184)	0.03 (0.02–0.04)	335 (273–395)	0.05 (0.04–0.06)	1.36 (1.23–1.49)
Southern Africa	62 (46–86)	0.05 (0.04–0.07)	128 (81–194)	0.07 (0.05–0.1)	0.81 (0.62–1.01)
Southern Latin America	32 (26–40)	0.07 (0.05–0.08)	59 (48–70)	0.09 (0.07–0.11)	1.32 (1.04–1.6)
Southern Sub-Saharan Africa	20 (15–25)	0.04 (0.03–0.05)	43 (34–51)	0.06 (0.05–0.07)	1.32 (1.1–1.55)
Sub-Saharan Africa—WB	239 (162–358)	0.03 (0.02–0.05)	678 (367–1,042)	0.05 (0.03–0.07)	1.34 (0.95–1.73)
Tropical Latin America	127 (112–142)	0.08 (0.07–0.09)	217 (189–245)	0.1 (0.09–0.12)	0.62 (0.31–0.92)
Western Africa	87 (26–139)	0.04 (0.01–0.06)	331 (101–544)	0.06 (0.03–0.1)	1.97 (1.53–2.4)
Western Europe	395 (376–416)	0.11 (0.11–0.12)	480 (426–532)	0.1 (0.09–0.11)	−0.17 (−0.36–0.02)
Western Pacific Region	539 (457–647)	0.04 (0.03–0.05)	1,424 (1,116–1,661)	0.07 (0.05–0.08)	2.26 (2.05–2.47)
Western Sub-Saharan Africa	89 (27–141)	0.03 (0.01–0.05)	333 (105–546)	0.06 (0.02–0.09)	1.86 (1.43–2.29)
World bank high income	926 (881–963)	0.1 (0.1–0.11)	1,208 (1,104–1,298)	0.09 (0.08–0.1)	−0.11 (−0.3–0.08)
World bank low income	117 (83–185)	0.02 (0.02–0.04)	247 (131–399)	0.03 (0.02–0.04)	0.55 (0.04–1.07)
World bank lower middle income	769 (542–1,015)	0.03 (0.02–0.05)	1,762 (1,311–2,197)	0.06 (0.04–0.07)	1.41 (1.24–1.59)
World bank upper middle Income	861 (730–1,022)	0.04 (0.04–0.05)	1,973 (1,628–2,229)	0.07 (0.06–0.08)	1.75 (1.57–1.92)

**Table 2:** The number of DALYs cases and the age-standardized DALYs rates attributable to neuroblastoma and other peripheral neuroblastoma in 1990 and 2021, and its trends from 1990 to 2021 globally.

Characteristics	1990		2021		1990–2021
	Number of DALYs cases(95% UI)	The age-standardized DALYs rate/100,000 (95% UI)	Number of DALYs cases(95% UI)	The age-standardized DALYs rate/100,000 (95% UI)	EAPC(95% CI)
Global	185,391 (158,570–219,544)	3.2 (2.75–3.78)	285,479 (227,709–341,110)	3.95 (3.11–4.77)	0.5 (0.41–0.59)
Sex					
Both	185,391 (158,570–219,544)	3.2 (2.75–3.78)	285,479 (227,709–341,110)	3.95 (3.11–4.77)	0.74 (0.65–0.82)
Female	83,547 (59,480–108,454)	2.94 (2.12–3.78)	119,905 (89,578–148,989)	3.37 (2.48–4.24)	0.55 (0.46–0.64)
Male	101,845 (88,714–115,086)	3.46 (3.02–3.9)	165,574 (129,629–203,435)	4.51 (3.48–5.6)	0.89 (0.8–0.97)
Age					
<5 years	107,017 (87,500–129,594)	17.26 (14.11–20.9)	122,165 (83,732–164,191)	18.56 (12.72–24.95)	0.43 (0.31–0.54)
10–14 years	8,581 (7,019–10,597)	1.6 (1.31–1.98)	14,294 (11,241–16,470)	2.14 (1.69–2.47)	0.95 (0.86–1.03)
15–19 years	5,118 (4,183–6,348)	0.99 (0.81–1.22)	8,727 (7,295–10,272)	1.4 (1.17–1.65)	0.97 (0.82–1.13)
5–9 years	29,459 (25,812–34,168)	5.05 (4.42–5.86)	37,727 (29,772–44,415)	5.49 (4.33–6.46)	0.43 (0.32–0.54)
SDI regions					
High-middle SDI	37,725 (31,790–44,161)	3.88 (3.27–4.54)	49,214 (40,524–55,695)	4.42 (3.61–5.11)	0.47 (0.31–0.63)
High SDI	45,979 (43,495–48,036)	6.44 (6.1–6.75)	44,718 (40,815–48,181)	5.25 (4.72–5.75)	−0.7 (−0.94–0.47)
Low-middle SDI	36,356 (27,689–48,440)	2.26 (1.74–2.98)	69,278 (51,418–89,166)	3.62 (2.69–4.65)	1.77 (1.61–1.93)
Low SDI	16,964 (11,363–26,823)	2.01 (1.35–3.2)	42,625 (23,605–67,375)	2.78 (1.58–4.36)	1.27 (0.86–1.67)
Middle SDI	48,187 (39,519–55,199)	2.54 (2.1–2.91)	79,412 (64,204–92,771)	3.72 (2.97–4.43)	1.31 (1.13–1.49)
GBD regions					
Advanced health system	68,394 (62,926–73,509)	6.22 (5.74–6.67)	64,569 (58,089–69,956)	5.34 (4.7–5.88)	−0.66 (−1–0.32)
Africa	24,207 (17,444–35,450)	2.44 (1.78–3.53)	59,461 (32,822–90,904)	3.34 (1.92–4.96)	1 (0.47–1.53)
African Region	20,974 (13,930–31,486)	2.55 (1.7–3.8)	54,834 (28,417–85,822)	3.57 (1.94–5.43)	1.11 (0.53–1.69)
America	40,162 (37,877–42,375)	5.42 (5.12–5.71)	46,368 (41,283–51,928)	5.2 (4.56–5.91)	−0.25 (−0.52–0.02)
Andean Latin America	1,865 (1,370–2,397)	3.98 (2.98–5.08)	2,417 (1,829–3,079)	3.78 (2.85–4.82)	−0.25 (−0.42–0.09)
Asia	81,218 (63,011–100,586)	2.3 (1.81–2.86)	142,519 (113,516–173,447)	3.59 (2.79–4.44)	1.16 (0.98–1.35)
Australasia	1,105 (988–1,237)	6.29 (5.58–7.08)	1,424 (1,174–1,715)	5.53 (4.41–6.89)	−0.51 (−0.8–0.21)
Basic Health System	60,763 (52,146–71,338)	2.48 (2.14–2.94)	98,936 (83,253–112,780)	3.49 (2.93–4.01)	1.01 (0.82–1.2)
Caribbean	1,616 (1,261–2,154)	4.15 (3.28–5.46)	2,654 (2,015–3,553)	6.31 (4.7–8.56)	1.16 (0.87–1.46)
Central Africa	955 (622–1,387)	0.91 (0.63–1.31)	1,307 (809–2,217)	0.66 (0.41–1.13)	−1.11 (−1.66–0.56)
Central Asia	815 (640–1,054)	1.12 (0.87–1.46)	2,039 (1,609–2,545)	2.15 (1.7–2.68)	2.86 (2.64–3.08)
Central Europe	4,750 (4,190–5,454)	4.42 (3.87–5.1)	4,172 (3,610–4,839)	3.96 (3.35–4.73)	−0.45 (−0.86–0.04)
Central Latin America	7,791 (7,183–8,553)	3.78 (3.5–4.12)	9,687 (8,064–11,547)	4.14 (3.41–5.02)	−0.21 (−0.62–0.21)
Central Sub-Saharan Africa	713 (350–1,282)	0.87 (0.52–1.41)	1,350 (956–1,899)	0.84 (0.59–1.21)	−0.21 (−0.71–0.29)
Commonwealth high income	7,926 (7,540–8,319)	8.88 (8.41–9.35)	6,645 (6,048–7,336)	5.78 (5.15–6.5)	−1.22 (−1.59–0.85)
Commonwealth low income	9,698 (6,839–14,413)	2.68 (1.93–4.01)	18,465 (9,868–29,008)	4.03 (2.17–6.3)	1 (0.43–1.58)
Commonwealth middle income	40,518 (25,046–55,679)	2.6 (1.6–3.57)	92,525 (63,198–124,548)	4.52 (3.09–6.08)	1.52 (1.22–1.83)
East Asia	22,928 (17,770–29,491)	2 (1.55–2.57)	43,531 (33,067–52,048)	3.29 (2.48–3.97)	1.86 (1.49–2.23)
East Asia & Pacific—WB	43,453 (35,914–52,714)	2.38 (1.97–2.89)	70,275 (56,744–80,466)	3.32 (2.68–3.85)	1.01 (0.77–1.25)
Eastern Africa	6,779 (4,463–12,419)	2.19 (1.44–3.92)	16,706 (8,760–28,117)	3.18 (1.68–5.23)	1.16 (0.48–1.84)
Eastern Europe	9,249 (6,714–12,289)	4.51 (3.34–5.95)	7,753 (6,603–8,825)	3.9 (3.23–4.51)	−1.18 (−1.48–0.87)
Eastern Mediterranean Region	12,821 (9,031–18,838)	2.29 (1.65–3.34)	35,205 (23,993–51,737)	4.31 (2.97–6.3)	2.18 (1.79–2.56)
Eastern Sub-Saharan Africa	10,483 (7,267–17,414)	3.04 (2.12–5.05)	23,354 (11,780–38,135)	3.94 (2.01–6.37)	0.81 (0.09–1.52)
Europe	39,495 (35,743–43,711)	5.87 (5.34–6.49)	36,792 (32,474–40,897)	5.34 (4.56–6.08)	−0.49 (−0.83–0.15)
Europe & Central Asia—WB	40,032 (36,203–44,338)	5.42 (4.93–6.01)	38,219 (33,701–42,394)	4.87 (4.17–5.49)	−0.49 (−0.78–0.19)
European Region	40,487 (36,695–44,785)	5.43 (4.95–6.01)	38,739 (34,165–42,986)	4.86 (4.17–5.48)	−0.49 (−0.78–0.2)
High-income Asia Pacific	9,093 (8,300–10,101)	7.2 (6.57–8.04)	7,546 (6,878–8,153)	6.49 (5.82–7.19)	−0.96 (−1.54–0.39)
High-income North America	17,162 (15,930–18,275)	7.3 (6.77–7.78)	16,296 (14,554–17,798)	5.53 (4.9–6.14)	−0.79 (−1.1–0.48)
Latin America & Caribbean—WB	23,131 (21,320–24,945)	4.53 (4.2–4.87)	30,178 (26,113–35,000)	5.07 (4.33–5.92)	0.07 (−0.25–0.39)
Limited health system	53,408 (36,219–73,741)	2.4 (1.62–3.33)	117,117 (78,121–159,173)	3.93 (2.64–5.32)	1.33 (0.96–1.7)
Middle East & North Africa—WB	6,327 (4,785–8,391)	1.8 (1.39–2.38)	9,792 (7,542–12,878)	2.13 (1.64–2.81)	0.3 (0.09–0.51)
Minimal health system	2,647 (1,772–4,298)	1.12 (0.76–1.8)	4,626 (2,455–8,409)	0.94 (0.51–1.7)	−0.52 (−1.22–0.18)
North Africa and Middle East	9,753 (7,033–13,678)	2.12 (1.56–2.95)	14,737 (11,234–18,934)	2.43 (1.86–3.13)	0.24 (0.02–0.46)
North America	17,160 (15,928–18,273)	7.3 (6.77–7.78)	16,296 (14,554–17,798)	5.53 (4.9–6.14)	−0.79 (−1.09–0.48)
Northern Africa	4,251 (3,107–5,949)	2.58 (1.92–3.59)	5,439 (3,904–7,800)	2.49 (1.79–3.57)	−0.38 (−0.64–0.11)
Oceania	27 (17–45)	0.42 (0.26–0.69)	48 (30–76)	0.36 (0.22–0.56)	−1.26 (−1.56–0.97)
Region of the Americas	40,162 (37,877–42,375)	5.42 (5.12–5.71)	46,368 (41,283–51,928)	5.2 (4.56–5.91)	−0.25 (−0.52–0.02)
South-East Asia Region	32,941 (23,152–44,208)	2.02 (1.42–2.7)	49,392 (37,997–61,369)	2.76 (2.09–3.47)	0.28 (0.06–0.51)
South Asia	34,571 (23,267–48,020)	2.35 (1.59–3.23)	65,630 (47,912–88,470)	3.97 (2.88–5.37)	1.24 (0.94–1.55)
South Asia—WB	35,104 (23,734–48,639)	2.34 (1.59–3.21)	66,479 (48,628–89,502)	3.86 (2.81–5.23)	1.2 (0.9–1.5)
Southeast Asia	10,825 (8,155–14,229)	2.03 (1.56–2.6)	18,475 (14,919–22,473)	2.9 (2.32–3.56)	0.68 (0.57–0.8)
Southern Africa	5,029 (3,631–7,090)	3.67 (2.74–5.11)	9,530 (5,660–15,160)	4.39 (2.71–6.78)	0.57 (0.15–0.99)
Southern Latin America	2,063 (1,670–2,595)	4.08 (3.31–5.11)	3,029 (2,430–3,739)	5.25 (4.11–6.67)	0.89 (0.57–1.21)
Southern Sub-Saharan Africa	1,368 (1,078–1,776)	2.27 (1.76–2.85)	2,481 (1,956–3,064)	3.21 (2.54–3.94)	0.98 (0.85–1.11)
Sub-Saharan Africa—WB	19,945 (13,515–30,138)	2.4 (1.62–3.6)	53,963 (27,847–85,351)	3.43 (1.86–5.27)	1.19 (0.6–1.79)
Tropical Latin America	9,852 (8,587–11,148)	5.84 (5.11–6.59)	12,483 (10,573–14,501)	6.34 (5.28–7.45)	0.02 (−0.3–0.35)
Western Africa	7,194 (2,065–11,290)	2.6 (0.8–4.14)	26,479 (7,149–44,868)	4.35 (1.35–7.09)	1.83 (1.21–2.45)
Western Europe	22,015 (20,997–23,121)	7.69 (7.32–8.09)	19,768 (17,394–22,326)	5.97 (5.16–6.94)	−0.79 (−1.2–0.38)
Western Pacific Region	37,220 (30,722–44,697)	2.52 (2.08–3.03)	59,771 (47,355–69,632)	3.51 (2.79–4.11)	1.08 (0.79–1.36)
Western Sub-Saharan Africa	7,348 (2,137–11,497)	2.39 (0.75–3.79)	26,606 (7,427–44,976)	3.88 (1.25–6.32)	1.72 (1.1–2.34)
World bank high income	55,170 (52,585–57,314)	6.84 (6.51–7.11)	51,918 (47,274–56,197)	5.54 (4.95–6.1)	−0.76 (−1.12–0.4)
World bank low income	9,966 (7,037–15,844)	1.75 (1.25–2.77)	20,101 (10,330–32,629)	2 (1.06–3.23)	0.39 (−0.26–1.05)
World bank lower middle income	60,874 (43,241–80,545)	2.41 (1.7–3.18)	124,119 (88,990–161,059)	3.84 (2.76–4.98)	1.23 (0.97–1.49)
World bank upper middle income	59,185 (50,531–69,118)	2.88 (2.45–3.36)	89,095 (74,694–100,880)	3.83 (3.21–4.37)	0.84 (0.59–1.09)

**Figure 1:**
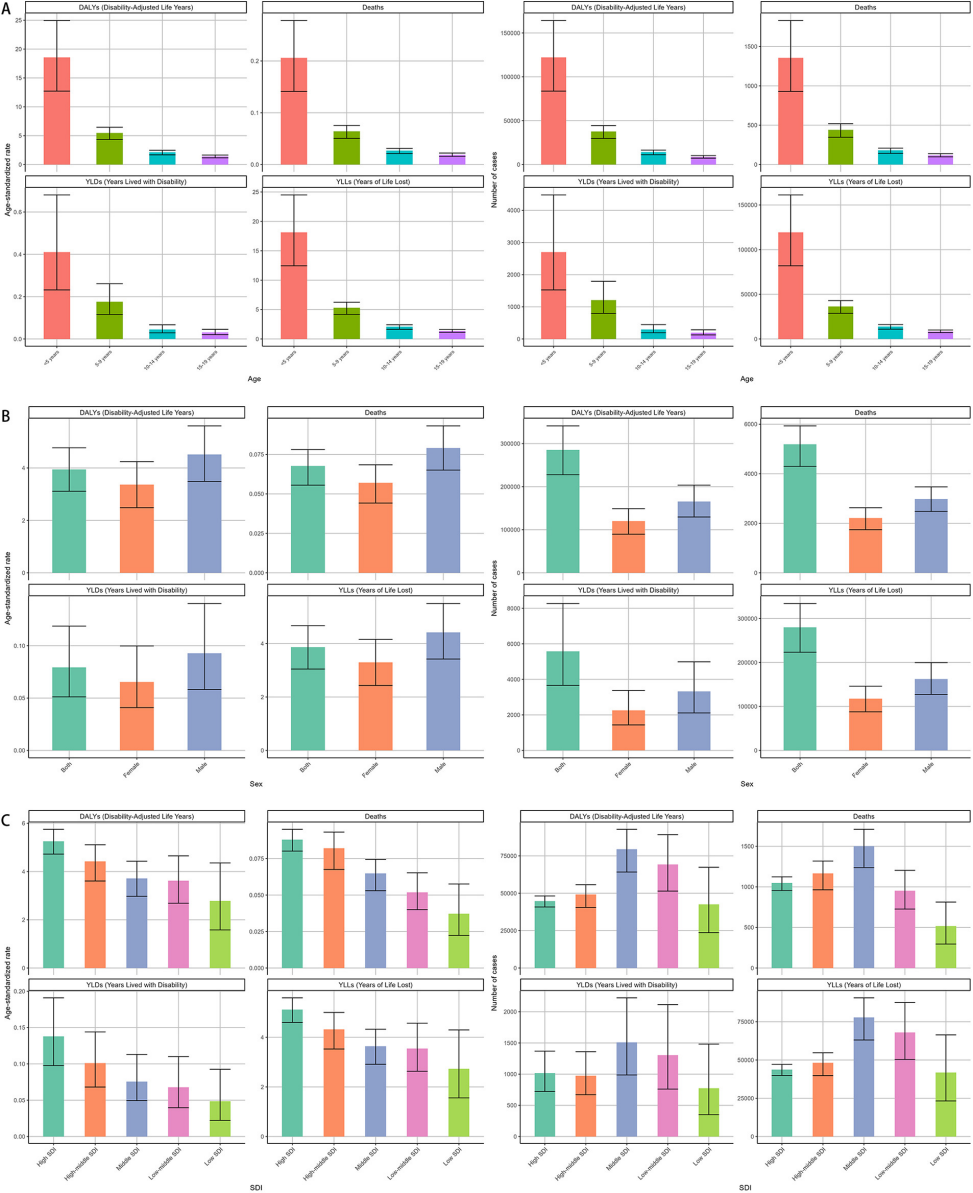
Global burden of NPNTs in 2021 by Sex, Age group, and SDI. **(A)** Numbers and ASRs of DALYs, deaths, YLDs, and YLLs by sex; **(B)** numbers and ASRs of DALYs, deaths, YLDs, and YLLs by age group; **(C)** numbers and ASRs of DALYs, deaths, YLDs, and YLLs by SDI quintiles. DALYs: disability-adjusted life years; YLDs: years lived with disability; YLLs: years of life lost; NPNTs: neuroblastoma and other peripheral nervous cell tumors; SDI: socio-demographic index.

Deaths and DALYs across age groups separately in 2021 were available in Figure 1. children under five years bore the highest burden of NPNTs, contributing 26.1% of global deaths (1,355/5,194) and 44.2% of DALYs (126,215/285,479). Their mortality rate (ASR: 0.21 per 100,000) was 10 times higher than adolescents aged 15–19 years (ASR: 0.02 per 100,000), with a similarly stark contrast in DALYs rates (18.56 vs. 1.4 per 100,000). Males experienced a 33% higher mortality rate than females (ASR: 0.08 vs. 0.06 per 100,000), accounting for 57% of deaths (2981 vs. 2213) and 58% of DALYs (165,574 vs. 119,905).

In 2021, middle-SDI regions bore the highest absolute burden of both deaths and DALYs due to neuroblastoma and other peripheral nervous cell tumors, with 1,503 deaths (95% CI: 1,238–1,710) and 79,412 DALYs (95% CI: 64,204–92,771). In contrast, high-SDI regions reported fewer deaths (1,048, 95% CI: 956–1,124) and DALYs (44,718, 95% CI: 40,815–48,181) but exhibited the highest age-standardized rates of both mortality (0.09 per 100,000, 95% CI: 0.08–0.10) and DALYs (5.25 per 100,000, 95% CI: 4.72–5.75), highlighting a disproportionate burden when adjusting for population age structure. (Figure 1, Tables 1, 2).

**Figure 2:**
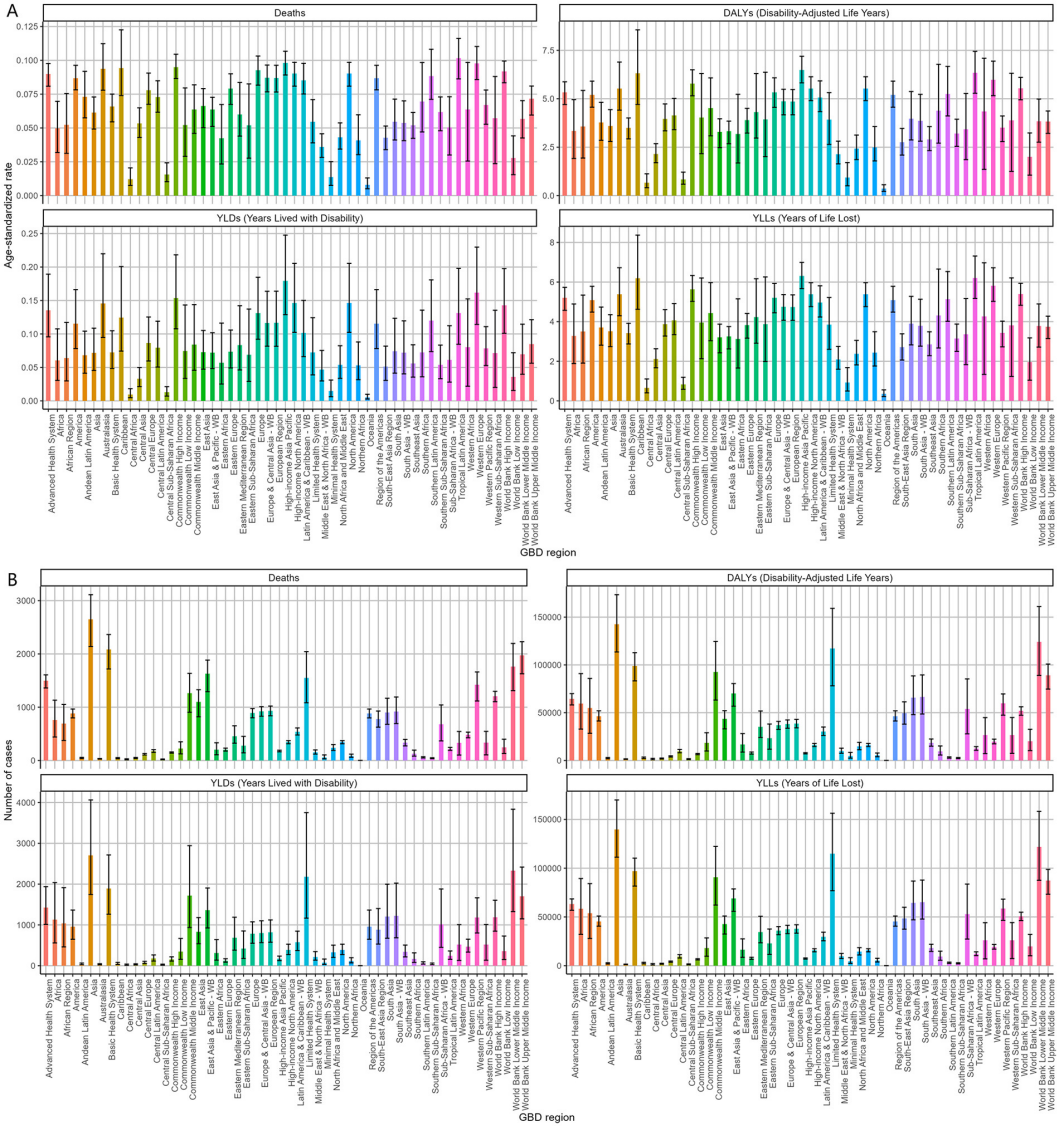
Regional burden of NPNTs in 2021. **(A)** ASRs of DALYs, deaths, YLDs, and YLLs across 21 GBD regions; **(B)** numbers of DALYs, deaths, YLDs, and YLLs across 21 GBD regions. DALYs: disability-adjusted life years; YLDs: years lived with disability; YLLs: years of life lost; NPNTs: neuroblastoma and other peripheral nervous cell tumors.

Asia exhibited the highest absolute burden of NPNTs, with 2,651 deaths and 142,519 DALYs in 2021. However, its age-standardized mortality rate (ASR: 0.06 per 100,000) was lower than in regions with advanced healthcare systems (e.g., the Advanced Health System region: ASR = 0.09 per 100,000), reflecting disparities in population size vs. healthcare efficiency. Notably, the Advanced Health System region recorded a higher DALYs rate (5.34 per 100,000) compared to Asia (3.59 per 100,000), suggesting unmet needs in managing long-term disability despite better survival outcomes. The Americas and Africa had comparable death counts (Americas: 882; Africa: 761), yet age-standardized mortality rates in the Americas were nearly twice as high (0.09 vs. 0.05 per 100,000). Southeast Asia exhibited the lowest DALYs rates (ASR: 2.9 per 100,000), while Trinidad and Tobago had the highest mortality globally (ASR: 0.24 per 100,000) (Figure 2, Tables 1, 2).

**Figure 3:**
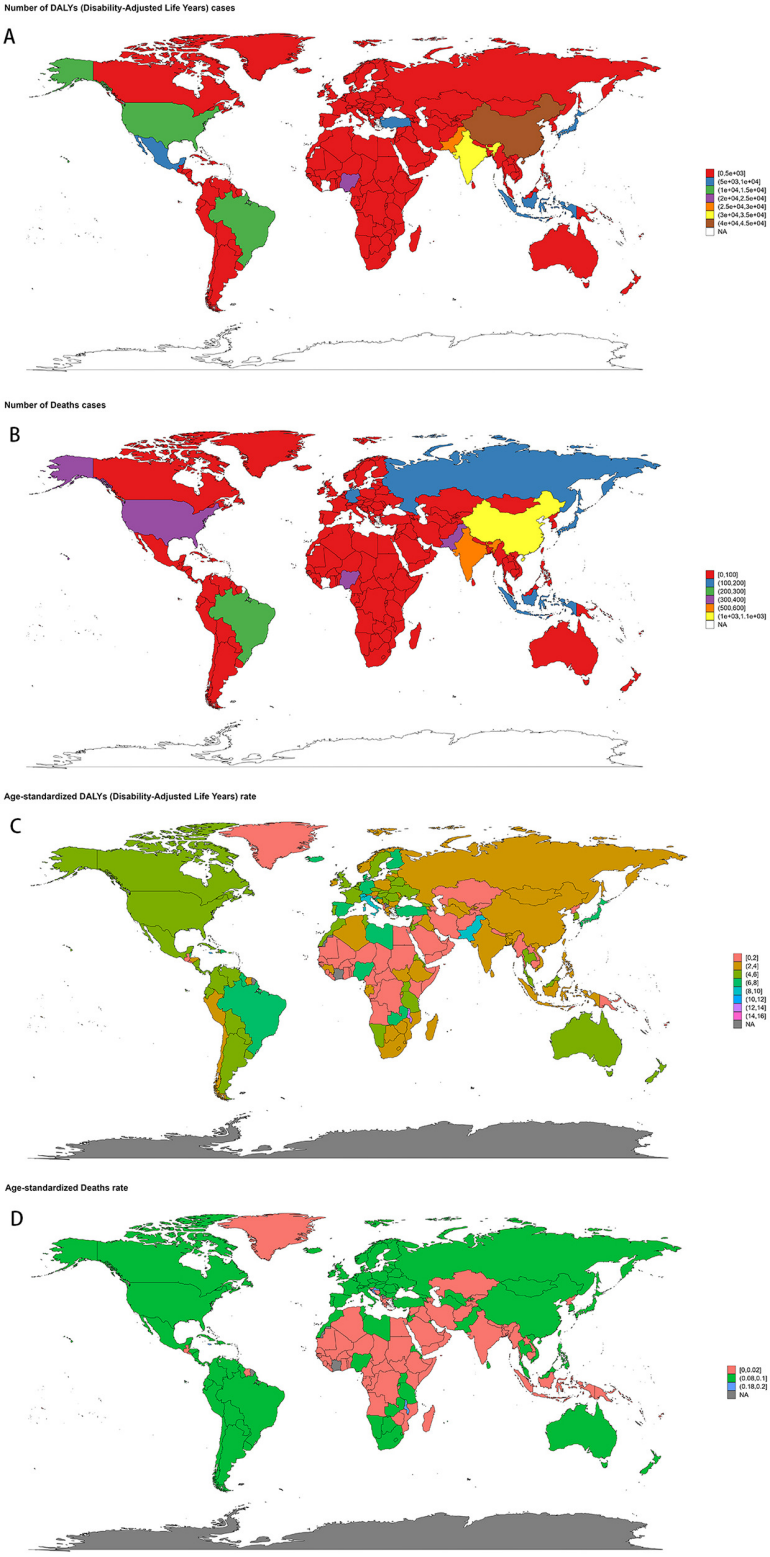
Geographic distribution of NPNTs burden in 2021. **(A)** numbers of DALYs; **(B)** numbers of deaths; **(C)** ASRs of DALYs; **(D)** ASRs of deaths. DALYs: disability-adjusted life years; NPNTs: neuroblastoma and other peripheral nervous cell tumors.

The disease burden varied significantly across the world (Figure 3). The highest age-standardized death rate (ASR) in 2021 was observed in Trinidad and Tobago (0.24, 95% UI: 0.18–0.31) per 100,000, followed by Barbados and Malta. The lowest ASR for deaths was observed in regions where the burden was negligible, including Afghanistan and Palau. The highest age-standardized DALYs rate (ASR) was also observed in Trinidad and Tobago (15.74, 95% UI: 11.54–20.96) per 100,000, followed by Barbados and Malawi. The lowest ASR for DALYs was in Tajikistan (0.05, 95% UI: 0.03–0.07), followed by Niger and Cabo Verde. As for the absolute numbers, the highest number of DALYs cases was observed in China (42,337, 95% UI: 31,944–50,792), followed by India (34,612, 95% UI: 25,595–44,339). Similarly, the highest number of deaths was recorded in China (1,070, 95% UI: 792–1,298), followed by India (519, 95% UI: 402–639). In contrast, many smaller countries and regions, such as Cabo Verde and Saint Kitts and Nevis, recorded a disease burden close to zero. Detailed data on NPNTs-related YLLs (Years of Life Lost) and YLDs (Years Lived with Disability), including both numbers and age-standardized rates across countries and territories in 2021, are available in Supplementary Figures S1.

### Age and sex patterns

**Figure 4:**
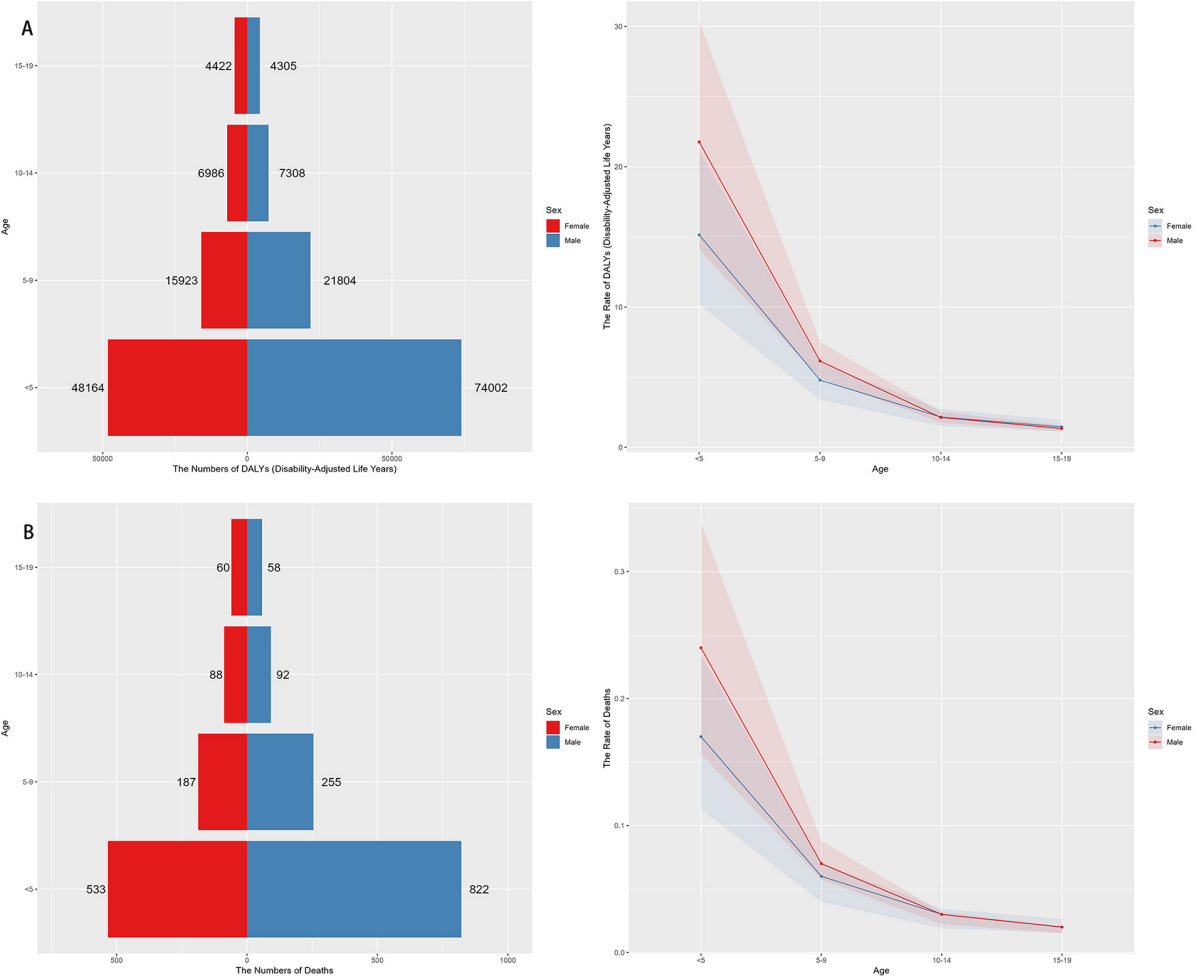
Sex- and Age-specific distribution of NPNTs burden in 2021. **(A)** numbers and ASRs of DALYs by sex and age group; **(B)** numbers and ASRs of deaths by sex and age group. DALYs: disability-adjusted life years; NPNTs: neuroblastoma and other peripheral nervous cell tumors.

In 2021, the highest global burden of neuroblastoma and other peripheral nervous cell tumors (NPNTs) in terms of DALYs and deaths was observed in children under the age of five, with both indicators decreasing progressively with age. Among children <5 years, males had a notably higher DALYs burden (74,002) compared to females (48,164). The number of DALYs declined significantly in the 5–9 and 10–14 age groups, with minimal values recorded in the 15–19 age group for both sexes. The number of deaths followed a similar trend, peaking in the <5 years age group, where males (822) exhibited higher mortality counts compared to females (533). Deaths continued to decrease across older age groups, with both sexes showing relatively small differences in the 10–14 and 15–19 age groups. The DALYs rates analysis further demonstrated a steep decline with increasing age, paralleling the observed death rate trends. The largest disparities in DALYs between sexes occurred in the <5 years group, where males exhibited significantly higher rates compared to females. Notably, males consistently showed higher DALYs and death rates across all age groups, reflecting a greater disease burden among males in early childhood (Figure 4). Detailed distributions of YLLs and YLDs are provided in Supplementary Figures S2.

### Temporal trend for neuroblastoma and other peripheral nervous cell tumors (NPNTs) from 1990 to 2021

**Figure 5:**
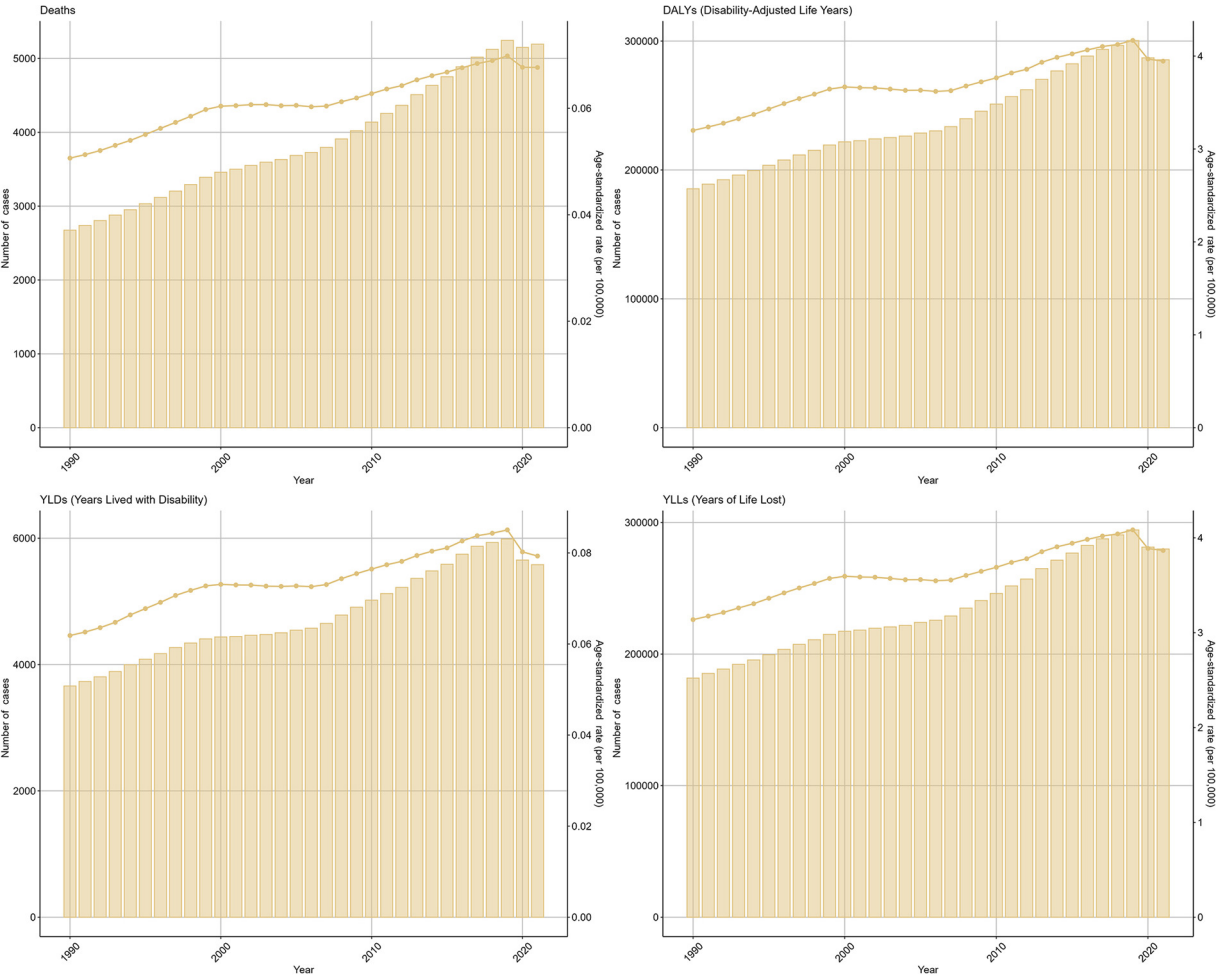
Global trends of NPNTs burden from 1990 to 2021. Numbers and ASRs of DALYs, deaths, YLDs, and YLLs. DALYs: disability-adjusted life years; YLDs: years lived with disability; YLLs: years of life lost; NPNTs: neuroblastoma and other peripheral nervous cell tumors.

The number of deaths due to Neuroblastoma and other Peripheral Nervous Cell Tumors (NPNTs) increased by 94.14% globally, from 2,675 cases in 1990 to 5,194 cases in 2021. Similarly, the number of DALYs increased by 53.97%, from 185,391 in 1990 to 285,479 in 2021. In contrast, the corresponding age-standardized death rate (ASR) and age-standardized DALYs rate (ASR) showed more modest increases, with the death ASR rising from 0.05 to 0.07 per 100,000 (EAPC: 0.93, 95% CI: 0.85–1.01) and the DALYs ASR increasing from 3.2 to 3.95 per 100,000 (EAPC: 0.50, 95% CI: 0.41–0.59). (Figure 5, Tables 1, 2 and Supplementary Tables S1, S2).

**Figure 6:**
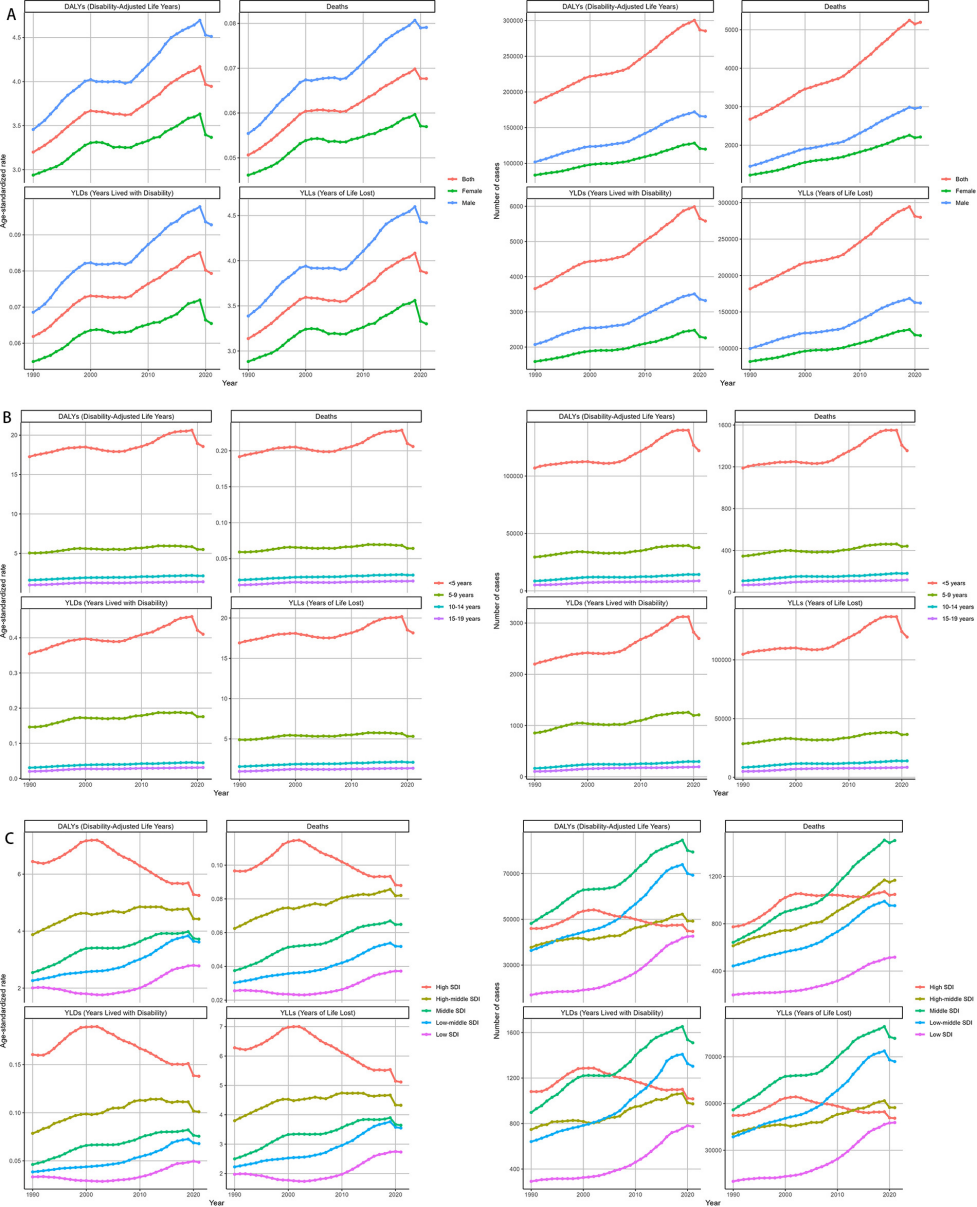
Temporal trends of NPNTs burden from 1990 to 2021 by Sex, Age, and SDI. **(A)** Numbers and ASRs of DALYs, deaths, YLDs, and YLLs by sex; **(B)** numbers and ASRs of DALYs, deaths, YLDs, and YLLs by age group; **(C)** numbers and ASRs of DALYs, deaths, YLDs, and YLLs by SDI quintiles. DALYs: disability-adjusted life years; YLDs: years lived with disability; YLLs: years of life lost; NPNTs: neuroblastoma and other peripheral nervous cell tumors; SDI: socio-demographic index.

From 1990 to 2021, Age-standardized rates (ASRs) of deaths and DALYs in males rose more sharply, peaking around 2019, while females exhibited a more gradual increase over the study period. The trends in males and females aligned with the overall upward trajectory but revealed a persistent gender disparity (Figure 6). Across age groups, the trends in deaths and DALYs due to Neuroblastoma and other Peripheral Nervous Cell Tumors (NPNTs) showed notable differences over time. Among children under five years old, both deaths and DALYs increased steadily until around 2019, followed by a clear decline. In the 5–9 years age group, deaths and DALYs exhibited a relatively stable trend with slight increases observed over the study period. For the 10–14 years and 15–19 years age groups, the trends remained largely flat, with minimal changes in both deaths and DALYs throughout the years (Figure 6).

At the SDI region level, trends in deaths and DALYs due to NPNTs varied substantially. In low-SDI and low-middle SDI regions, both the number of deaths and DALYs, as well as their age-standardized rates (ASRs), exhibited consistent increases over time. In contrast, high-SDI regions demonstrated a stable or declining trend in ASRs of deaths and DALYs, despite a slight initial increase in absolute numbers. The high-middle SDI region showed relative stability in trends, whereas the middle-SDI region experienced a substantial increase in burden until around 2019, followed by a slight decline (Figure 6).

**Figure 7:**
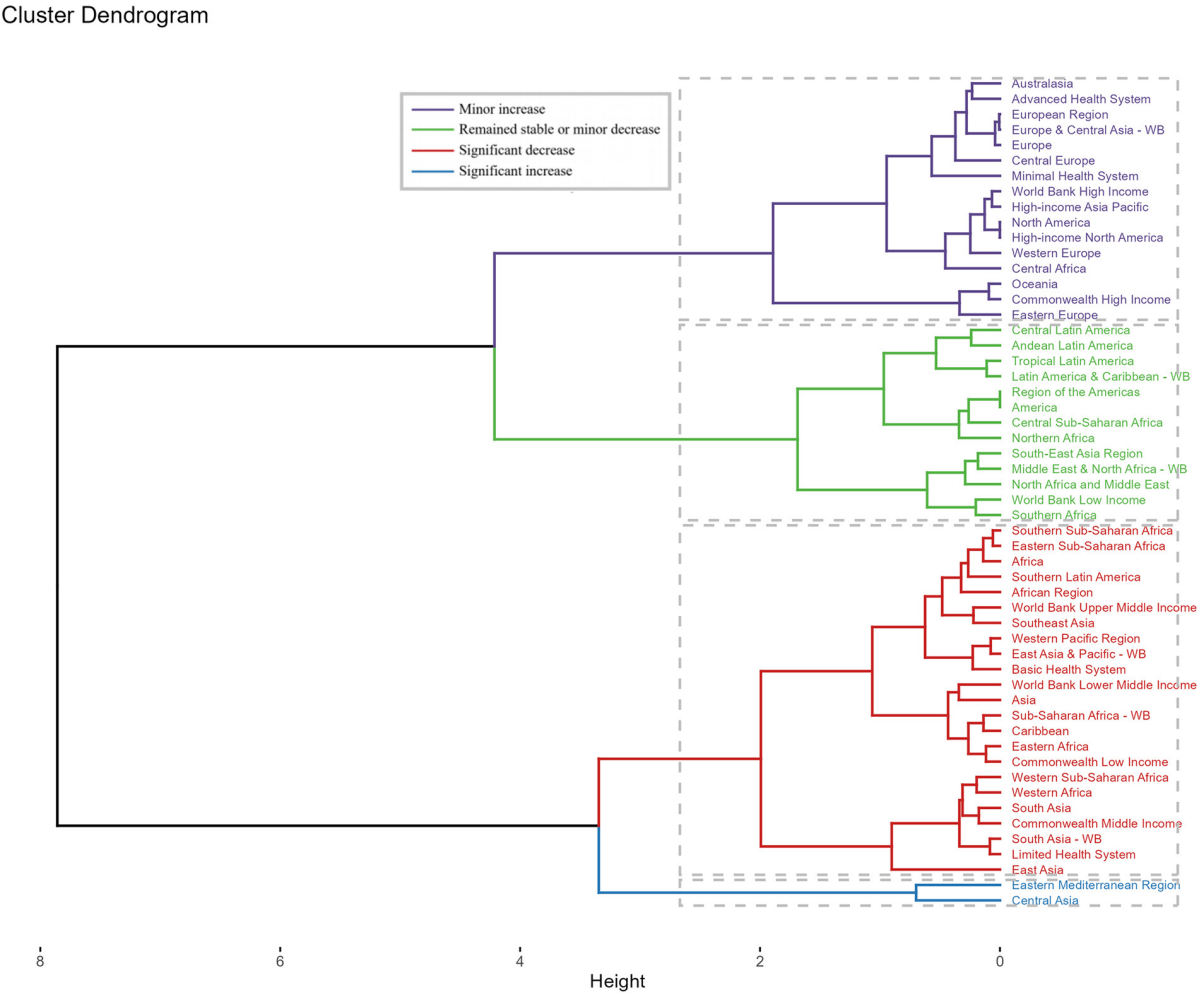
Cluster analysis based on EAPCs of NPNTs burden. Clusters of countries according to the EAPCs of ASRs for DALYs and deaths from 1990 to 2021. EAPC: estimated annual percentage change; NPNTs: neuroblastoma and other peripheral nervous cell tumors.

Across GBD regions, the disease burden of Neuroblastoma and other Peripheral Nervous Cell Tumors (NPNTs) showed distinct patterns. A hierarchical clustering analysis revealed that regions such as Central Asia and the Eastern Mediterranean Region experienced significant increases in both deaths and DALYs rates. In contrast, regions including Sub-Saharan Africa, Southeast Asia, and Asia demonstrated significant decreases in these rates over the study period (Figure 7).

Across countries and territories where the number of deaths and DALYs was non-zero in both 1990 and 2021, the most pronounced increase in the number of deaths cases from 1990 to 2021 was observed in the Dominican Republic (350.0%), followed by Chile (333.33%) and Australia (73.33%). The most pronounced decrease in deaths was observed in Portugal (deaths: −16.67%), followed by Republic of Korea (deaths: −15.79%). The most pronounced increase in the number of DALYs cases was observed in Guyana (1,400%), followed by Georgia (1,160%). The most pronounced decrease in DALYs cases was observed in Greenland (−66.67%) and Liberia (−66.67%). For ASRs, the largest increase in deaths and DALYs burden from 1990 to 2021 was observed in Georgia [deaths: EAPC = 13.74, 95% confidence interval (CI) 12.72–14.76; DALYs: EAPC = 13.35, 95% CI 12.41–14.29], followed by Guyana [deaths: EAPC = 7.89, 95% CI 6.22–9.58; DALYs: EAPC = 7.74, 95% CI 6.05–9.46]. The largest decrease occurred in Niger [deaths: EAPC = −5.21, 95% CI −5.79–4.62; DALYs: EAPC = −6.54, 95% CI −7.25–5.83], followed by Liberia [deaths: EAPC = −3.97, 95% CI −4.68–3.26; DALYs: EAPC = −5.36, 95% CI −6.14–4.58] (Supplementary Figure S3). While this study focuses on the trends in deaths and DALYs, detailed data on YLDs (Years Lived with Disability) and YLLs (Years of Life Lost) are provided in the Supplementary Materials (Supplementary Figure S4).

### Decomposition analysis of DALYs and deaths for neuroblastoma and other peripheral nervous cell tumors (NPNTs) from 1990 to 2021

**Figure 8:**
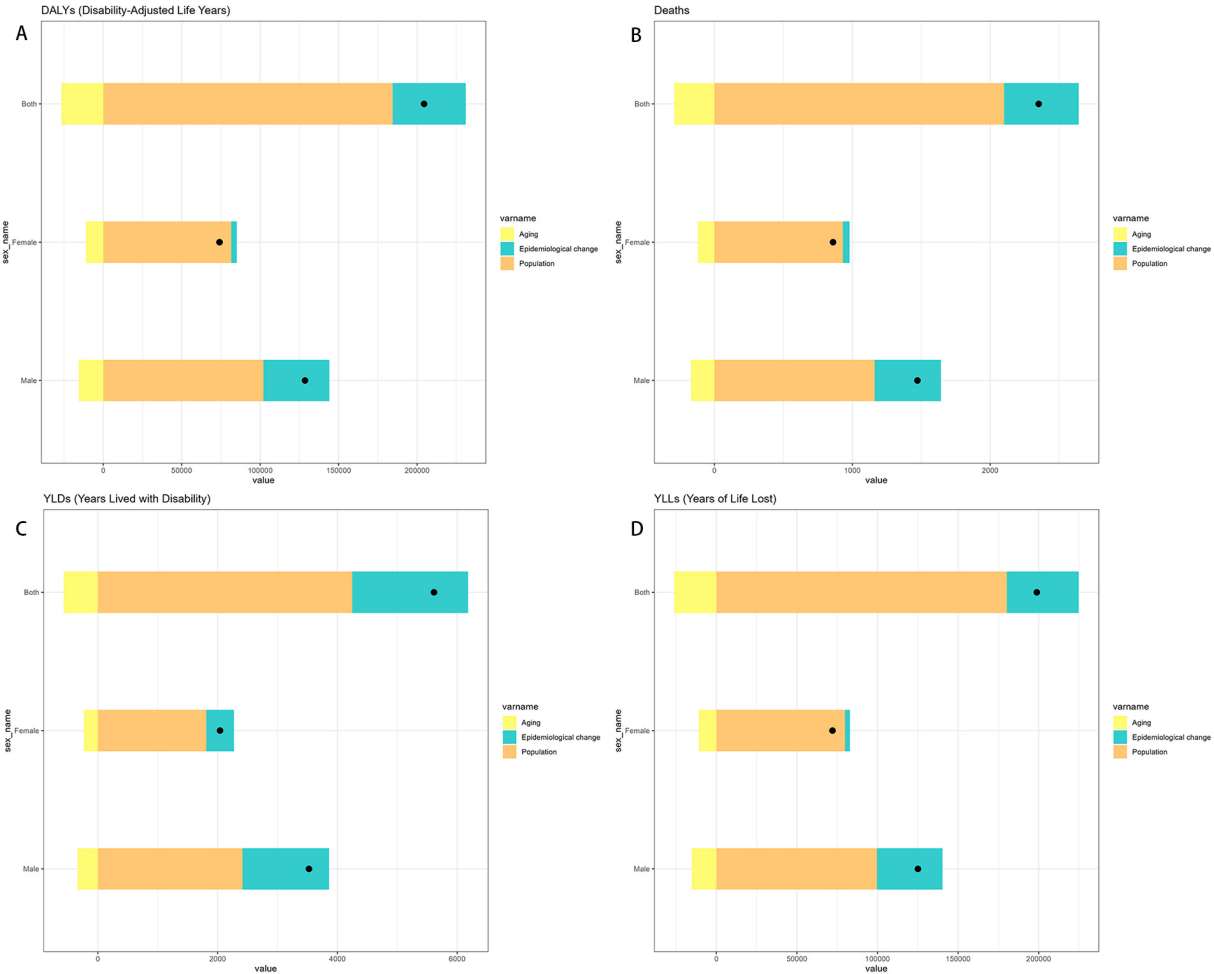
Decomposition of change in NPNTs burden by Sex (1990–2021). **(A)** decomposition of DALYs; **(B)** decomposition of deaths; **(C)** decomposition of YLDs; **(D)** decomposition of YLLs. DALYs: disability-adjusted life years; YLDs: years lived with disability; YLLs: years of life lost; NPNTs: neuroblastoma and other peripheral nervous cell tumors.

By performing decomposition analysis on the DALYs and deaths attributable to Neuroblastoma and other peripheral nervous cell tumors (NPNTs), this study evaluated the impact of factors such as aging, population growth, and epidemiological changes from 1990 to 2021 (Figure 8 and Table 3). Globally, the overall DALYs difference showed an upward trend, with population growth being the predominant contributor (90.23%), followed by epidemiological changes (22.79%), while aging had a negative contribution (−13.02%).For deaths, a similar trend was observed globally, with the overall deaths difference driven primarily by population growth (89.38%), followed by epidemiological changes (22.98%), while aging had a smaller negative contribution (−12.36%).

**Table 3:** Decomposition of changes in the number of DALYs and deaths, YLLs, YLDs for NPNTs by sex (1990–2021).

Characteristics	Sex	Overall difference	Aging	Population	Epidemiological change	Percent change of aging	Percent change of population	Percent change of epidemiological change	The DALYs value in 1990	The DALYs value in 2021	The net change in DALYs from 1990 to 2021
DALYs	Male	128,592.88	−15,573.772	102,127.295	42,039.36	−12.11	79.42	32.69	101,844.79	165,573.9	63,729.09
	Female	74,115.82	−10,997.479	81,622.973	3,490.322	−14.84	110.13	4.71	83,546.61	119,904.7	36,358.13
	Both	204,437.64	−26,626.81	184,463.37	46,601.085	−13.02	90.23	22.79	185,391.4	285,478.6	100,087.21
Deaths	Male	1,472.36	−170.676	1,162.112	480.922	−11.59	78.93	32.66	1,451.929	2,980.661	1,528.7322
	Female	860.22	−119.442	932.151	47.507	−13.89	108.36	5.52	1,222.835	2,213.149	990.3135
	Both	2,352	−290.74	2,102.272	540.464	−12.36	89.38	22.98	2,674.764	5,193.81	2,519.0457
YLDs	Male	3,525.2	−336.952	2,416.752	1,445.395	−9.56	68.56	41	2,072.323	3,320.863	1,248.5408
	Female	2,039.8	−232.572	1,811.276	461.1	−11.4	88.8	22.61	1,588.8	2,260.756	671.9567
	Both	5,613.12	−571.01	4,248.948	1,935.184	−10.17	75.7	34.48	3,661.122	5,581.62	1,920.4975
YLLs	Male	125,067.69	−15,236.82	99,710.542	40,593.965	−12.18	79.73	32.46	99,772.47	162,253	62,480.55
	Female	72,076.01	−10,764.907	79,811.697	3,029.222	−14.94	110.73	4.2	81,957.81	117,644	35,686.17
	Both	198,824.52	−26,055.799	180,214.422	44,665.901	−13.1	90.64	22.46	181,730.28	279,897	98,166.72

When stratified by sex, the contributions of the three factors exhibited distinct patterns. Among males, the overall DALYs difference increased by 128,592.88, primarily driven by population growth (79.42%) and epidemiological changes (32.69%), with aging contributing negatively (−12.11%). In contrast, for females, the DALYs increase of 74,115.82 was predominantly driven by population growth (110.13%), while epidemiological changes had a minor contribution (4.71%) and aging showed a more pronounced negative impact (−14.84%).For deaths, the trends were similar but less pronounced. Among males, population growth contributed 78.93%, epidemiological changes 32.66%, and aging −11.59%. For females, population growth accounted for 108.36% of the increase, whereas epidemiological changes contributed only 5.52%, and aging had a negative impact of −13.89%.

Overall, population growth emerged as the dominant driver of the increasing burden of NPNTs globally, while aging consistently offset part of the increase. These trends varied by sex, with females exhibiting a stronger influence of population growth relative to males, while epidemiological changes played a more prominent role among males.

### Predicted trends for DALYs and deaths of neuroblastoma and other peripheral nervous cell tumors (NPNTs) (2022–2050)

**Figure 9:**
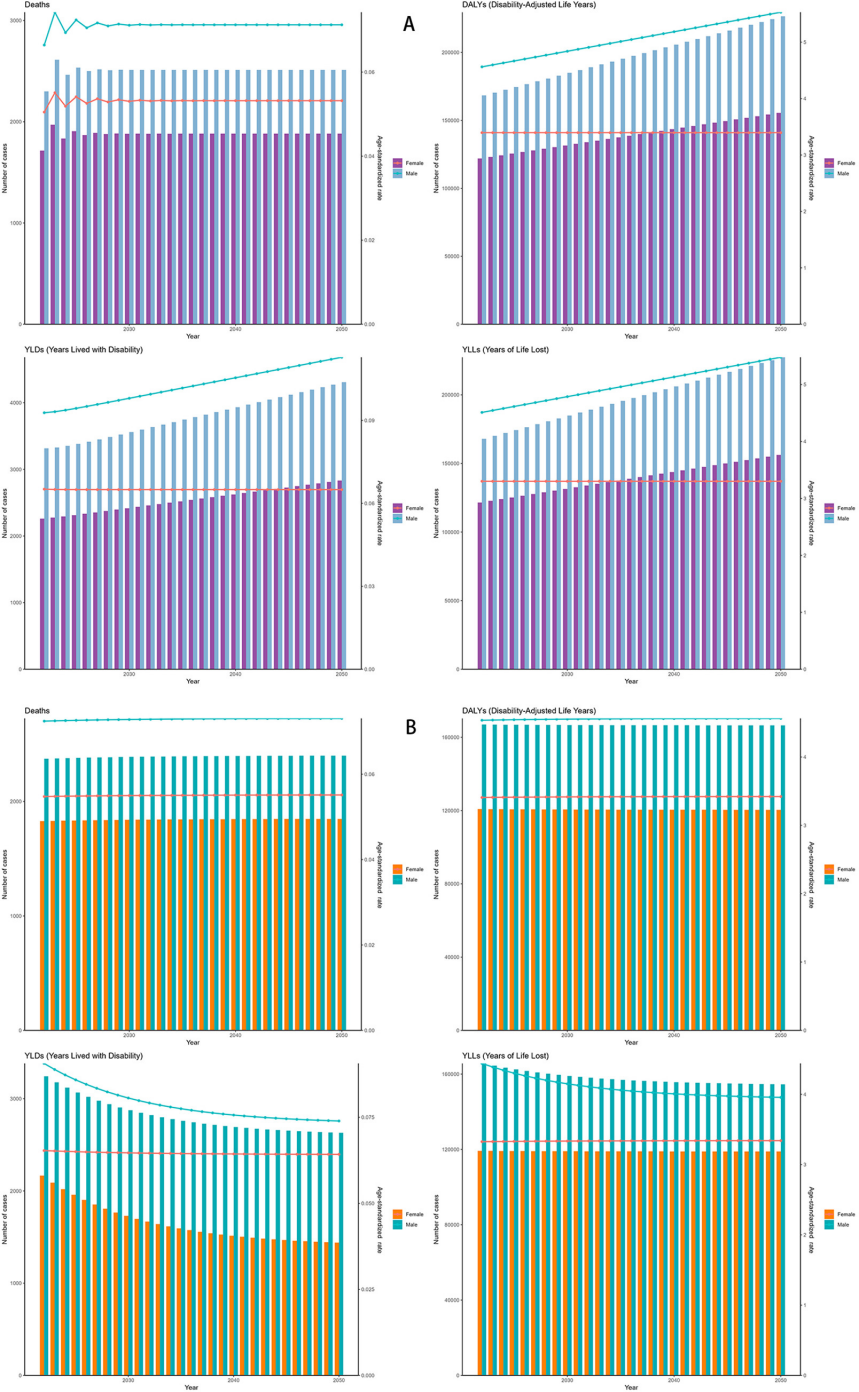
Forecasted trends in NPNTs burden (2022–2050). **(A)** ARIMA model predictions fornumbers and ASRs of DALYs and deaths; **(B)** ES model predictions for numbers and ASRs of DALYs and deaths. DALYs: disability-adjusted life years; ARIMA: autoregressive integrated moving average; ES: exponential smoothing; NPNTs: neuroblastoma and other peripheral nervous cell tumors.

Both the ES and ARIMA models indicated an increase in the number of DALYs and deaths attributable to NPNTs from 2022 to 2050. However, the trends and magnitude of change varied between the two models. The ES model predicted that the total number of DALYs would remain stable for both males and females, with minimal changes in ASRs. In contrast, the ARIMA model forecasted a gradual increase in DALYs, particularly among males, with ASRs showing a noticeable upward trend. For example, the male DALYs under the ES model stabilized at around 166,000, while the ARIMA model predicted an increase to 226,533 by 2050. Similarly, male ASRs under the ES model remained relatively constant (from 4.54 to 4.56), while the ARIMA model projected a significant rise (from 4.56 to 5.53) (Figure 9, Table 4).

**Table 4:** Comparison of DALYs and deaths predictions between ES and ARIMA models (2022–2050).

Indicator	ES model prediction	ARIMA model prediction
Male DALYs	2022: 166,953 (95% CI: 158,689–175,216)	2022: 168,345 (95% CI: 160,849–175,841)
	2050: 166,514 (95% CI: 84,439–248,590)	2050: 226,533 (95% CI: 186,164–266,902)
Female DALYs	2022: 120,843 (95% CI: 114,933–126,753)	2022: 121,916 (95% CI: 116,424–127,407)
	2050: 120,393 (95% CI: 73,262–167,524)	2050: 155,449 (95% CI: 125,917–185,062)
Male deaths	2022: 2,373 (95% CI: 1,665–3,081)	2022: 2,300 (95% CI: 1,617–2,983)
	2050: 2,400 (95% CI: 814–3,985)	2050: 2,511 (95% CI: −18–5,041)
Female deaths	2022: 1,827 (95% CI: 1,363–2,292)	2022: 1,715 (95% CI: 1,271–2,159)
	2050: 1,847 (95% CI: 841–2,854)	2050: 1,882 (95% CI: 294–3,470)
Male ASR (deaths)	2022: 0.0724 (95% CI: 0.0623–0.0825)	2022: 0.0664 (95% CI: 0.0570–0.0759)
	2050: 0.0730 (95% CI: 0.0577–0.0833)	2050: 0.0712 (95% CI: 0.0390–0.1034)
Female ASR (deaths)	2022: 0.0548 (95% CI: 0.0495–0.0608)	2022: 0.0505 (95% CI: 0.0458–0.0551)
	2050: 0.0551 (95% CI: 0.0471–0.0630)	2050: 0.0532 (95% CI: 0.0379–0.0684)
Male ASR (DALYs)	2022: 4.54 (95% CI: 4.33–4.75)	2022: 4.56 (95% CI: 4.36–4.77)
	2050: 4.56 (95% CI: 3.68–5.45)	2050: 5.53 (95% CI: 4.44–6.62)
Female ASR (DALYs)	2022: 3.41 (95% CI: 3.25–3.56)	2022: 3.40 (95% CI: 3.24–3.55)
	2050: 3.42 (95% CI: 2.80–4.05)	2050: 3.40 (95% CI: 2.56–4.22)

For deaths, both models suggested stable trends in the number of cases and ASRs. However, ARIMA predicted a slightly higher increase in male deaths compared to the ES model. By 2050, male deaths under the ES model stabilized at 2,400, while the ARIMA model forecasted an increase to 2,511.63. The corresponding ASRs for males showed minimal changes in the ES model, while ARIMA indicated a slight upward trend (Figure 9 and Table 4). Notably, the ARIMA model's prediction interval for male deaths in 2050 included a slightly negative lower bound. This arises from symmetric statistical estimation applied to low baseline values and should be interpreted as reflecting near-zero burden, rather than indicating actual negative mortality.

## Discussion

The global burden of Neuroblastoma and other Peripheral Nervous Cell Tumors (NPNTs) in 2021 of this study provides essential insights into their epidemiology and public health implications, particularly in the context of global health and pediatric oncology, where early diagnosis and effective treatment can significantly improve survival rates and reduce long-term morbidity. The data revealed substantial variability in mortality and morbidity rates across different age groups, genders, and socio-demographic regions, reflecting the multifaceted nature of these malignancies and their associated challenges. For instance, the disproportionately high burden observed in children under five years old aligns with established patterns in pediatric oncology, emphasizing their vulnerability due to developmental and biological factors (11). This is supported by findings indicating that neuroblastoma accounts for approximately 15% of pediatric cancer-related deaths, particularly affecting younger children due to their developmental stage (12). The significantly lower rates in adolescents suggest a distinct age-related susceptibility, potentially influenced by biological differences and delayed progression in older age groups. These findings align with observations that age-dependent tumor biology plays a key role in modulating disease outcome (13). The highly aggressive nature of neuroblastoma, including its rapid progression in early childhood, has been associated in previous studies with MYCN amplification, a well-established marker of poor prognosis (14).

The observed gender disparity, with males bearing a higher burden than females, adds another layer of complexity, pointing toward potential genetic, hormonal, or environmental contributors. This pronounced advantage in mortality and DALYs among males may be attributed to a combination of genetic predispositions, such as MYCN amplification, physiological factors like androgen influence on tumor microenvironments, and socio-cultural factors including disparities in healthcare-seeking behaviors (15, 16). The male predominance in NPNTs may stem from synergistic interactions between hormonal signaling and oncogenic drivers. Some experimental studies have proposed a mechanism in which DHT upregulates MYCN expression through androgen receptor binding, potentially contributing to male predominance, while simultaneously inhibiting apoptosis through BCL−2 activation (14). Previous studies (11, 17) have highlighted the role of androgens in modulating tumor microenvironments, which could partially explain the higher burden among males. These differences underscore the need for further exploration of sex-specific mechanisms in disease development and progression, particularly regarding interactions between genetic predispositions and hormonal influences.

Regional disparities in disease burden are pronounced, particularly across socio-demographic index (SDI) regions. Middle SDI regions exhibit the highest mortality and disability-adjusted life year rates, reflecting limited healthcare access and resources. For instance, limited availability of early diagnostic tools and specialized pediatric oncology centers in these regions often leads to delayed diagnosis and treatment, exacerbating mortality and morbidity rates (18). In contrast, high-SDI regions, despite recording lower disease burdens, benefit from advanced healthcare systems, early diagnosis, and better treatment options. These findings reaffirm the inequities in global healthcare infrastructure and the urgent need for targeted interventions in resource-limited settings (19). We propose two priority actions including implement urine catecholamine metabolite screening in high-burden regions to enable early detection of neuroblastoma and centralize pediatric oncology expertise in regional hubs while training primary care providers in low-SDI regions to recognize early symptoms.

Significant variation in disease burden was observed across GBD regions. Asia bore the highest burden, particularly in terms of deaths and disability-adjusted life years (DALYs). This pattern aligns with findings noting that population size and uneven healthcare access contribute significantly to the Asian burden of NPNTs (20). Advanced Health System regions and the Americas also showed substantial disease burdens, albeit influenced by more advanced healthcare systems compared to low-resource settings such as parts of Africa and Southeast Asia.Globally, the disease burden varied significantly. Countries such as Trinidad and Tobago exhibited the highest age-standardized death and DALY rates, while regions with stronger healthcare infrastructure, like parts of Southeast Asia and the Americas, showed relatively lower rates. Larger countries like China and India recorded the highest death and DALY counts, reflecting the impact of population size alongside healthcare challenges. Conversely, smaller nations with negligible burdens further illustrate how national healthcare capacities and reporting systems contribute to observed discrepancies (21).

The age and sex patterns observed in the global burden of NPNTs further emphasize the critical need for targeted interventions. Children under five years bore the highest burden, with males experiencing disproportionately higher disability-adjusted life years (DALYs) and death rates compared to females. This aligns with findings that emphasize the heightened biological vulnerabilities of younger children to aggressive tumor types like neuroblastoma (22, 23). The progressive decline in DALYs and deaths across older age groups reflects an age-dependent reduction in vulnerability, corroborating findings suggesting that tumor biology and immune system maturation play significant roles in these differences (3).

Decomposition analysis provides further insights into the factors driving the observed changes in NPNT burden. Population growth emerged as the dominant contributor to increases in both deaths and DALYs, accounting for the majority of the observed differences. This aligns with findings emphasizing the role of demographic shifts in shaping disease trends (24). Epidemiological changes also played a significant role, particularly among males, where their influence was more pronounced. Conversely, aging exerted a negative impact, offsetting part of the overall increase. So, in high-fertility regions (e.g., Sub-Saharan Africa), scaling pediatric oncology capacity proportionally to population growth is critical, while in middle-SDI regions, targeted investments in early screening and MYCN amplification diagnostics could amplify the mitigating effects of epidemiological changes.

Predicted trends for DALYs and deaths from 2022 to 2050 further underscore the complex interplay of demographic and epidemiological factors. Morgenstern et al. (2016) emphasized that demographic factors like population growth and healthcare disparities remain critical in shaping long-term trends in pediatric cancers, including neuroblastoma. The ES model predicts relatively stable trends for both sexes, reflecting gradual improvements in healthcare delivery and disease management (25). In contrast, the ARIMA model forecasts a sharper rise, particularly among males, highlighting potential challenges in addressing persistent disparities. These findings align with analyses by Hwang et al. (26), which suggested that higher tumor mutational burdens in males may contribute to their disproportionately rising DALYs. By integrating predictive insights from both models, future health policies can better address the disparities and complexities of managing NPNTs across different populations. These findings reinforce the need for dynamic, sex-specific public health strategies to mitigate the growing burden of NPNTs globally.

The findings of this study underscore critical public health implications for addressing the burden of Neuroblastoma and other Peripheral Nervous Cell Tumors (NPNTs). Some studies (2021) noted that demographic and healthcare inequalities are major drivers of pediatric cancer disparities globally (7, 27). Priority interventions should focus on strengthening early diagnostic capabilities and expanding access to specialized pediatric oncology care, particularly in low- and middle-SDI regions where resource constraints exacerbate disparities. Establishing cost-effective screening programs in high-burden regions, guided by risk-based approaches, could significantly improve early detection and treatment outcomes. Gerstl et al. (28) highlighted the importance of policy-level collaborations in bridging healthcare gaps and improving cancer outcomes, particularly for resource-limited populations. At a global level, fostering international collaborations to share resources and expertise in pediatric oncology is essential. Investments in research to explore genetic and molecular underpinnings of neuroblastoma, such as MYCN amplification, could inform the development of more effective, personalized therapies. These strategies, in conjunction with strengthening healthcare infrastructure and fostering equity in resource allocation, are critical for reducing the global burden of NPNTs and achieving better health outcomes for all affected populations.

## Limitations

This study provides critical insights into the global burden of NPNTs, but several limitations must be acknowledged. While the GBD 2021 database offers comprehensive and standardized estimates, it relies on ecological modeling techniques and indirect statistical inference, rather than individual-level data. This methodology may obscure regional heterogeneity, especially in low- and middle-income countries LMICs where cancer registries are sparse or underdeveloped. The quality, completeness, and timeliness of primary data sources vary widely across regions, leading to potential underreporting in pediatric populations, particularly in conflict-affected settings (e.g., Afghanistan) and rural areas. These limitations may result in underestimated mortality and DALYs and affect cross-regional comparisons. Moreover, this study did not investigate biological mechanisms such as MYCN amplification or androgen signaling.

Additionally, our predictive models (Exponential Smoothing and ARIMA) are based on historical trends and assume continuity, without accounting for unexpected global events or technological breakthroughs. Disruptions such as global pandemics or the emergence of MYCN-targeted therapies could substantially alter future trajectories, a limitation inherent to all long-term forecasting approaches. Furthermore, although our decomposition analysis successfully quantified the effects of population growth and aging, it could not fully disentangle coexisting epidemiological dynamics such as improved diagnostics, changing treatment protocols, or delays in care. Future studies integrating primary surveillance data, high-resolution registry inputs, and scenario-based forecasting models may improve precision and mitigate these constraints.

## Conclusion

This study underscores the substantial global burden of NPNTs, particularly among children under five years and males, who bear disproportionately higher mortality and DALY rates. The findings reveal significant regional disparities, highlighting the pressing need for equitable healthcare access and targeted interventions.

Addressing these challenges requires a multifaceted approach. Strengthening diagnostic and therapeutic capabilities in middle-SDI regions is paramount, alongside investments in genetic and molecular research, such as exploring the role of MYCN amplification in neuroblastoma, to support the development of personalized treatment approaches. International collaborations should also be fostered to share expertise and resources. By addressing these disparities and leveraging predictive insights, policymakers can reduce global inequities and improve outcomes for affected populations worldwide.

## Data Availability

Publicly available datasets were analyzed in this study. This data can be found here: https://vizhub.healthdata.org/gbd-results.
