# Supplementary material for: Global burden, trends, and projections to 2050 of neuroblastoma and other peripheral nervous cell tumors: a systematic analysis of the global burden of disease study from 1990 to 2021
Source: Front Pediatr. 2025 Sep 3;13:1604053. doi: 10.3389/fped.2025.1604053 (PMC12440315; doi:10.3389/fped.2025.1604053)
Supplement: Supplementary file 1 [file Table1.docx]

**Table S1. The number of deaths cases and age-standardized rates of Neuroblastoma and other peripheral neuroblastoma deaths across countries and territories in 1990 and 2021, and its trends from 1990 to 2021 globally.**

| Characteristics | 1990 | | 2021 | | 1990–2021 |
| --- | --- | --- | --- | --- | --- |
|  | Number of deaths  cases(95% UI) | The age-standardized deaths  rate/100000(95% UI) | Number of deaths  cases(95% UI) | The age-standardized deaths  rate/100000(95% UI) | EAPC(95% CI) |
| Albania | 1 (0-1) | 0.02 (0.01-0.02) | 1 (0-1) | 0.03 (0.01-0.04) | 0.76 (0.48-1.05) |
| Algeria | 15 (9-23) | 0.04 (0.03-0.07) | 18 (13-27) | 0.04 (0.03-0.06) | -0.62 (-0.76--0.49) |
| American Samoa | 0 (0-0) | 0.02 (0.01-0.04) | 0 (0-0) | 0.02 (0.01-0.04) | -0.48 (-1.14-0.19) |
| Andorra | 0 (0-0) | 0.12 (0.08-0.18) | 0 (0-0) | 0.07 (0.05-0.11) | -0.34 (-0.62--0.05) |
| Angola | 2 (1-4) | 0.01 (0.01-0.03) | 7 (4-11) | 0.02 (0.01-0.03) | 0.98 (0.72-1.25) |
| Antigua and Barbuda | 0 (0-0) | 0.05 (0.03-0.07) | 0 (0-0) | 0.07 (0.05-0.1) | 1.2 (0.95-1.46) |
| Argentina | 26 (21-33) | 0.08 (0.06-0.1) | 41 (33-50) | 0.09 (0.08-0.12) | 0.93 (0.69-1.16) |
| Armenia | 1 (0-1) | 0.03 (0.02-0.05) | 4 (2-6) | 0.1 (0.06-0.14) | 5.9 (5.41-6.39) |
| Australia | 15 (14-17) | 0.1 (0.09-0.11) | 26 (21-31) | 0.09 (0.08-0.11) | -0.08 (-0.26-0.1) |
| Austria | 7 (7-8) | 0.1 (0.09-0.12) | 7 (6-9) | 0.07 (0.05-0.09) | -0.85 (-1.08--0.63) |
| Azerbaijan | 2 (1-3) | 0.03 (0.02-0.05) | 3 (2-5) | 0.03 (0.02-0.05) | 1.28 (0.89-1.67) |
| Bahamas | 0 (0-0) | 0.08 (0.06-0.1) | 0 (0-0) | 0.1 (0.07-0.14) | 0.76 (0.53-0.98) |
| Bahrain | 0 (0-0) | 0.02 (0.01-0.03) | 1 (0-1) | 0.06 (0.03-0.08) | 4.58 (3.91-5.25) |
| Bangladesh | 37 (22-61) | 0.02 (0.01-0.04) | 53 (30-81) | 0.03 (0.02-0.05) | 0.44 (0.1-0.78) |
| Barbados | 0 (0-1) | 0.19 (0.15-0.23) | 1 (0-1) | 0.21 (0.15-0.29) | 0.72 (0.59-0.85) |
| Belarus | 8 (6-10) | 0.08 (0.06-0.1) | 16 (12-21) | 0.12 (0.09-0.16) | 1.54 (1.26-1.82) |
| Belgium | 9 (7-11) | 0.1 (0.08-0.12) | 11 (9-13) | 0.09 (0.07-0.11) | -0.28 (-0.51--0.05) |
| Belize | 0 (0-0) | 0.08 (0.06-0.09) | 0 (0-1) | 0.1 (0.08-0.13) | 0.74 (0.13-1.34) |
| Benin | 1 (0-1) | 0.01 (0-0.01) | 0 (0-1) | 0 (0-0.01) | -3.49 (-3.98--2.98) |
| Bermuda | 0 (0-0) | 0.03 (0.03-0.04) | 0 (0-0) | 0.05 (0.04-0.06) | 1.66 (1.21-2.1) |
| Bhutan | 0 (0-0) | 0.02 (0.01-0.04) | 0 (0-0) | 0.03 (0.01-0.06) | 1.53 (1.28-1.79) |
| Bolivia (Plurinational State of) | 5 (3-7) | 0.06 (0.04-0.09) | 9 (6-13) | 0.08 (0.06-0.12) | 0.46 (0.33-0.58) |
| Bosnia and Herzegovina | 1 (1-2) | 0.03 (0.02-0.05) | 2 (1-3) | 0.05 (0.03-0.07) | 2.48 (2.21-2.75) |
| Botswana | 0 (0-0) | 0.02 (0.01-0.04) | 1 (1-2) | 0.06 (0.04-0.08) | 2.77 (2.45-3.09) |
| Brazil | 125 (110-139) | 0.08 (0.07-0.09) | 213 (186-241) | 0.1 (0.09-0.12) | 0.61 (0.3-0.92) |
| Brunei Darussalam | 0 (0-0) | 0.08 (0.05-0.12) | 0 (0-0) | 0.08 (0.06-0.1) | -0.25 (-0.5-0.01) |
| Bulgaria | 3 (2-5) | 0.04 (0.03-0.06) | 5 (3-7) | 0.06 (0.04-0.09) | 0.6 (0.15-1.05) |
| Burkina Faso | 1 (0-1) | 0 (0-0.01) | 1 (0-1) | 0 (0-0) | -2.89 (-3.38--2.41) |
| Burundi | 3 (2-5) | 0.03 (0.02-0.06) | 4 (1-10) | 0.02 (0.01-0.05) | -0.59 (-1.21-0.03) |
| Cabo Verde | 0 (0-0) | 0 (0-0) | 0 (0-0) | 0 (0-0) | 4.95 (4.6-5.31) |
| Cambodia | 2 (1-3) | 0.01 (0.01-0.02) | 4 (2-6) | 0.02 (0.01-0.04) | 1.28 (1.1-1.47) |
| Cameroon | 1 (1-2) | 0.01 (0.01-0.01) | 2 (1-3) | 0.01 (0-0.01) | -2.67 (-3.11--2.23) |
| Canada | 27 (23-30) | 0.11 (0.09-0.13) | 35 (30-42) | 0.09 (0.07-0.12) | -0.39 (-0.52--0.26) |
| Central African Republic | 0 (0-1) | 0.01 (0.01-0.02) | 1 (0-1) | 0.01 (0.01-0.02) | 0 (-0.12-0.12) |
| Chad | 0 (0-1) | 0 (0-0.01) | 0 (0-1) | 0 (0-0) | -2.67 (-3.14--2.2) |
| Chile | 3 (3-4) | 0.02 (0.02-0.03) | 13 (11-16) | 0.07 (0.05-0.09) | 3.62 (2.88-4.37) |
| China | 318 (254-406) | 0.03 (0.02-0.04) | 1070 (792-1298) | 0.07 (0.05-0.08) | 3.33 (3.01-3.66) |
| Colombia | 19 (15-23) | 0.06 (0.05-0.07) | 38 (29-48) | 0.08 (0.06-0.1) | 0.96 (0.5-1.43) |
| Comoros | 0 (0-1) | 0.04 (0.02-0.07) | 1 (0-1) | 0.07 (0.04-0.12) | 0.93 (0.5-1.35) |
| Congo | 1 (0-1) | 0.03 (0.02-0.04) | 2 (1-2) | 0.03 (0.02-0.05) | 0.47 (0.3-0.63) |
| Cook Islands | 0 (0-0) | 0.01 (0-0.01) | 0 (0-0) | 0.01 (0.01-0.02) | 2.06 (1.87-2.25) |
| Costa Rica | 2 (2-3) | 0.07 (0.05-0.08) | 4 (3-5) | 0.08 (0.07-0.1) | 0.36 (0.19-0.54) |
| Côte d'Ivoire | 2 (1-4) | 0.01 (0-0.02) | 5 (2-10) | 0.01 (0.01-0.03) | 0.19 (-0.36-0.73) |
| Croatia | 5 (4-6) | 0.1 (0.08-0.12) | 9 (7-12) | 0.15 (0.11-0.2) | 1.61 (1.28-1.94) |
| Cuba | 8 (6-10) | 0.08 (0.06-0.1) | 10 (8-12) | 0.09 (0.07-0.11) | 1.26 (0.9-1.63) |
| Cyprus | 1 (0-1) | 0.09 (0.06-0.13) | 1 (1-2) | 0.09 (0.07-0.12) | 0.6 (0.34-0.87) |
| Czechia | 8 (7-11) | 0.08 (0.06-0.11) | 14 (10-20) | 0.1 (0.07-0.13) | 0.7 (0.37-1.04) |
| Democratic People's Republic of Korea | 6 (4-10) | 0.03 (0.02-0.05) | 8 (5-12) | 0.03 (0.02-0.05) | 0.21 (0.05-0.36) |
| Democratic Republic of the Congo | 6 (3-10) | 0.01 (0.01-0.02) | 9 (5-15) | 0.01 (0.01-0.02) | -0.61 (-1.04--0.18) |
| Denmark | 3 (2-3) | 0.05 (0.04-0.06) | 8 (6-9) | 0.11 (0.08-0.14) | 1.78 (1.43-2.13) |
| Djibouti | 0 (0-1) | 0.06 (0.03-0.1) | 1 (0-2) | 0.07 (0.03-0.12) | 0.12 (-0.24-0.48) |
| Dominica | 0 (0-0) | 0.05 (0.03-0.07) | 0 (0-0) | 0.14 (0.08-0.23) | 3.16 (2.87-3.44) |
| Dominican Republic | 2 (1-5) | 0.03 (0.02-0.06) | 9 (6-13) | 0.09 (0.06-0.12) | 2.33 (1.79-2.88) |
| Ecuador | 4 (3-6) | 0.04 (0.04-0.06) | 15 (12-20) | 0.09 (0.07-0.12) | 2.74 (2.35-3.12) |
| Egypt | 12 (6-26) | 0.02 (0.01-0.04) | 23 (15-44) | 0.02 (0.02-0.05) | 0.44 (0.1-0.77) |
| El Salvador | 2 (1-2) | 0.03 (0.02-0.03) | 2 (2-3) | 0.03 (0.03-0.05) | 0.47 (0.33-0.61) |
| Equatorial Guinea | 0 (0-0) | 0.01 (0.01-0.02) | 1 (0-1) | 0.04 (0.03-0.07) | 3.52 (3.27-3.78) |
| Eritrea | 2 (1-3) | 0.03 (0.02-0.05) | 4 (2-8) | 0.06 (0.03-0.1) | 1.59 (1.12-2.06) |
| Estonia | 1 (1-2) | 0.08 (0.06-0.12) | 3 (2-4) | 0.13 (0.09-0.19) | 1.63 (1.21-2.05) |
| Eswatini | 0 (0-0) | 0.03 (0.02-0.04) | 0 (0-1) | 0.05 (0.03-0.08) | 2.2 (1.88-2.51) |
| Ethiopia | 20 (9-63) | 0.03 (0.01-0.07) | 53 (23-136) | 0.04 (0.02-0.09) | 1.11 (0.57-1.64) |
| Fiji | 0 (0-0) | 0.04 (0.02-0.05) | 0 (0-1) | 0.03 (0.02-0.07) | -1.43 (-1.99--0.87) |
| Finland | 3 (2-3) | 0.05 (0.04-0.06) | 9 (7-11) | 0.13 (0.11-0.17) | 3.18 (2.77-3.58) |
| France | 53 (47-59) | 0.1 (0.09-0.11) | 65 (53-81) | 0.09 (0.07-0.11) | -0.11 (-0.26-0.05) |
| Gabon | 0 (0-0) | 0.03 (0.02-0.04) | 1 (0-1) | 0.04 (0.03-0.06) | 1.45 (1.32-1.59) |
| Gambia | 0 (0-1) | 0.02 (0.01-0.03) | 1 (0-1) | 0.03 (0.01-0.05) | -0.14 (-0.66-0.39) |
| Georgia | 0 (0-1) | 0.01 (0-0.01) | 6 (4-8) | 0.12 (0.08-0.16) | 13.74 (12.72-14.76) |
| Germany | 74 (63-88) | 0.09 (0.08-0.11) | 104 (86-125) | 0.1 (0.08-0.13) | 0.38 (0.12-0.64) |
| Ghana | 0 (0-1) | 0 (0-0.01) | 1 (0-1) | 0 (0-0) | -2.53 (-3.45--1.6) |
| Greece | 4 (4-5) | 0.04 (0.04-0.05) | 6 (5-6) | 0.05 (0.04-0.05) | 0.96 (0.75-1.16) |
| Greenland | 0 (0-0) | 0.08 (0.05-0.1) | 0 (0-0) | 0.04 (0.02-0.05) | -1.81 (-2.01--1.6) |
| Grenada | 0 (0-0) | 0.04 (0.03-0.06) | 0 (0-0) | 0.08 (0.06-0.1) | 1.93 (1.68-2.18) |
| Guam | 0 (0-0) | 0.01 (0.01-0.01) | 0 (0-0) | 0.01 (0.01-0.02) | 2.8 (2.03-3.59) |
| Guatemala | 2 (2-4) | 0.02 (0.02-0.03) | 3 (2-4) | 0.02 (0.02-0.02) | -1.04 (-1.27--0.8) |
| Guinea | 3 (1-5) | 0.03 (0.01-0.05) | 6 (2-12) | 0.03 (0.01-0.06) | -0.1 (-0.77-0.59) |
| Guinea-Bissau | 0 (0-0) | 0.01 (0-0.01) | 0 (0-0) | 0 (0-0.01) | -3.17 (-3.58--2.75) |
| Guyana | 0 (0-0) | 0 (0-0.01) | 1 (0-1) | 0.09 (0.06-0.12) | 7.89 (6.22-9.58) |
| Haiti | 4 (2-8) | 0.05 (0.02-0.09) | 9 (5-16) | 0.07 (0.04-0.11) | 1.12 (0.81-1.44) |
| Honduras | 2 (1-3) | 0.03 (0.02-0.05) | 5 (3-7) | 0.06 (0.04-0.08) | 0.98 (0.79-1.17) |
| Hungary | 9 (8-11) | 0.09 (0.07-0.1) | 14 (11-19) | 0.11 (0.08-0.15) | 0.88 (0.43-1.33) |
| Iceland | 0 (0-0) | 0.08 (0.07-0.1) | 0 (0-1) | 0.11 (0.08-0.14) | 1.47 (1.25-1.69) |
| India | 285 (172-394) | 0.03 (0.02-0.04) | 519 (402-639) | 0.04 (0.03-0.05) | 0.6 (0.35-0.85) |
| Indonesia | 42 (28-62) | 0.02 (0.02-0.03) | 108 (83-137) | 0.04 (0.03-0.06) | 1.81 (1.65-1.96) |
| Iran (Islamic Republic of) | 3 (1-4) | 0 (0-0.01) | 12 (3-16) | 0.02 (0-0.02) | 5.05 (4.57-5.54) |
| Iraq | 5 (3-8) | 0.02 (0.01-0.03) | 16 (11-24) | 0.04 (0.03-0.06) | 2.04 (1.81-2.27) |
| Ireland | 4 (3-4) | 0.1 (0.09-0.12) | 4 (3-5) | 0.07 (0.06-0.09) | -1 (-1.21--0.78) |
| Israel | 5 (4-6) | 0.1 (0.08-0.13) | 8 (6-10) | 0.08 (0.06-0.1) | -0.81 (-1.05--0.57) |
| Italy | 59 (54-63) | 0.14 (0.13-0.15) | 75 (63-85) | 0.13 (0.1-0.15) | 0.34 (0.04-0.63) |
| Jamaica | 2 (1-2) | 0.07 (0.05-0.08) | 4 (3-5) | 0.16 (0.1-0.22) | 2.37 (1.79-2.94) |
| Japan | 95 (91-98) | 0.1 (0.1-0.1) | 141 (130-152) | 0.11 (0.1-0.12) | 0.43 (0.06-0.8) |
| Jordan | 2 (1-3) | 0.05 (0.03-0.07) | 7 (5-10) | 0.06 (0.05-0.09) | 0.49 (0.3-0.68) |
| Kazakhstan | 3 (2-4) | 0.02 (0.01-0.03) | 6 (4-9) | 0.03 (0.02-0.05) | 1.78 (1.59-1.96) |
| Kenya | 5 (3-9) | 0.02 (0.01-0.03) | 14 (9-21) | 0.03 (0.02-0.04) | 1.87 (1.49-2.25) |
| Kiribati | 0 (0-0) | 0 (0-0) | 0 (0-0) | 0 (0-0) | 0.98 (0.75-1.21) |
| Kuwait | 1 (1-1) | 0.04 (0.03-0.05) | 2 (1-2) | 0.05 (0.04-0.07) | 3.41 (2.06-4.78) |
| Kyrgyzstan | 2 (1-2) | 0.03 (0.02-0.04) | 2 (2-3) | 0.04 (0.03-0.05) | 5.66 (4.19-7.15) |
| Lao People's Democratic Republic | 1 (0-1) | 0.01 (0.01-0.02) | 2 (1-2) | 0.02 (0.01-0.04) | 1.95 (1.81-2.09) |
| Latvia | 2 (1-2) | 0.06 (0.04-0.09) | 2 (1-3) | 0.07 (0.05-0.09) | 1.61 (1.18-2.03) |
| Lebanon | 1 (1-1) | 0.03 (0.02-0.04) | 2 (2-3) | 0.04 (0.03-0.07) | 1.42 (1.29-1.54) |
| Lesotho | 0 (0-0) | 0.01 (0.01-0.03) | 1 (0-1) | 0.03 (0.02-0.05) | 3.07 (2.77-3.36) |
| Liberia | 0 (0-0) | 0.01 (0-0.01) | 0 (0-0) | 0 (0-0.01) | -3.97 (-4.68--3.26) |
| Libya | 3 (2-5) | 0.05 (0.03-0.09) | 6 (4-9) | 0.11 (0.06-0.16) | 1.86 (1.54-2.18) |
| Lithuania | 2 (1-3) | 0.06 (0.04-0.08) | 5 (3-6) | 0.11 (0.08-0.15) | 3.22 (2.87-3.57) |
| Luxembourg | 0 (0-0) | 0.1 (0.09-0.11) | 0 (0-0) | 0.06 (0.05-0.07) | -1.48 (-1.66--1.31) |
| Madagascar | 8 (5-13) | 0.04 (0.03-0.06) | 15 (8-24) | 0.04 (0.02-0.07) | -0.18 (-0.78-0.42) |
| Malawi | 24 (15-35) | 0.13 (0.09-0.19) | 39 (16-77) | 0.16 (0.07-0.3) | 0.35 (-0.34-1.05) |
| Malaysia | 9 (5-12) | 0.05 (0.03-0.07) | 27 (21-32) | 0.09 (0.07-0.11) | 1.83 (1.59-2.08) |
| Maldives | 0 (0-1) | 0.08 (0.04-0.16) | 1 (0-1) | 0.14 (0.11-0.2) | 1.17 (0.81-1.53) |
| Mali | 2 (0-3) | 0.01 (0-0.02) | 4 (1-10) | 0.01 (0.01-0.02) | -0.29 (-1.01-0.44) |
| Malta | 1 (1-1) | 0.18 (0.15-0.22) | 1 (1-1) | 0.19 (0.15-0.26) | 0.44 (0.3-0.59) |
| Marshall Islands | 0 (0-0) | 0.01 (0-0.01) | 0 (0-0) | 0.01 (0.01-0.01) | 1.27 (0.87-1.68) |
| Mauritania | 0 (0-0) | 0.01 (0.01-0.01) | 0 (0-0) | 0.01 (0-0.01) | -2.85 (-3.27--2.42) |
| Mauritius | 0 (0-0) | 0.03 (0.02-0.03) | 1 (1-1) | 0.07 (0.06-0.08) | 3.64 (3.03-4.25) |
| Mexico | 52 (48-57) | 0.05 (0.05-0.06) | 91 (79-103) | 0.07 (0.06-0.09) | 0.54 (0.08-1.01) |
| Micronesia (Federated States of) | 0 (0-0) | 0.01 (0-0.01) | 0 (0-0) | 0.01 (0.01-0.01) | 0.27 (-0.01-0.55) |
| Monaco | 0 (0-0) | 0 (0-0) | 0 (0-0) | 0 (0-0) | 0.31 (0.04-0.57) |
| Mongolia | 0 (0-1) | 0.03 (0.02-0.04) | 2 (1-2) | 0.06 (0.04-0.07) | 2.69 (2.29-3.09) |
| Montenegro | 0 (0-0) | 0.05 (0.04-0.08) | 0 (0-1) | 0.05 (0.04-0.07) | 0.95 (0.79-1.11) |
| Morocco | 16 (11-23) | 0.05 (0.04-0.07) | 26 (17-38) | 0.07 (0.05-0.11) | 1.01 (0.86-1.17) |
| Mozambique | 8 (5-16) | 0.04 (0.02-0.07) | 18 (7-41) | 0.04 (0.02-0.09) | 0.46 (-0.22-1.14) |
| Myanmar | 7 (4-12) | 0.02 (0.01-0.03) | 17 (12-23) | 0.03 (0.02-0.04) | 1.86 (1.76-1.96) |
| Namibia | 1 (0-1) | 0.04 (0.02-0.05) | 2 (1-3) | 0.08 (0.05-0.11) | 2.22 (2.06-2.39) |
| Nauru | 0 (0-0) | 0.01 (0.01-0.02) | 0 (0-0) | 0.01 (0.01-0.02) | 0.17 (-0.19-0.53) |
| Nepal | 5 (3-8) | 0.02 (0.01-0.03) | 7 (3-11) | 0.02 (0.01-0.04) | 0.61 (0.32-0.91) |
| Netherlands | 19 (17-22) | 0.14 (0.12-0.16) | 22 (18-27) | 0.1 (0.08-0.13) | -0.85 (-1.12--0.58) |
| New Zealand | 4 (3-4) | 0.12 (0.1-0.14) | 5 (5-6) | 0.09 (0.08-0.11) | -0.04 (-0.45-0.37) |
| Nicaragua | 4 (3-6) | 0.08 (0.06-0.11) | 4 (3-6) | 0.07 (0.06-0.1) | -0.97 (-1.19--0.75) |
| Niger | 1 (0-1) | 0 (0-0.01) | 0 (0-1) | 0 (0-0) | -5.21 (-5.79--4.62) |
| Nigeria | 76 (22-123) | 0.06 (0.02-0.1) | 312 (90-523) | 0.11 (0.04-0.18) | 2.18 (1.75-2.61) |
| Niue | 0 (0-0) | 0.01 (0.01-0.02) | 0 (0-0) | 0.03 (0.02-0.03) | 1.33 (1.06-1.61) |
| North Macedonia | 1 (1-1) | 0.05 (0.04-0.06) | 1 (1-2) | 0.05 (0.03-0.06) | 0.71 (0.53-0.9) |
| Northern Mariana Islands | 0 (0-0) | 0 (0-0) | 0 (0-0) | 0 (0-0) | 1.63 (0.94-2.32) |
| Norway | 4 (4-4) | 0.09 (0.09-0.1) | 6 (5-7) | 0.08 (0.07-0.09) | -1.1 (-1.48--0.72) |
| Oman | 1 (0-1) | 0.02 (0.01-0.05) | 1 (1-2) | 0.04 (0.02-0.05) | 0.81 (0.42-1.19) |
| Pakistan | 92 (60-142) | 0.06 (0.04-0.08) | 322 (214-484) | 0.12 (0.08-0.18) | 2.73 (2.31-3.16) |
| Palau | 0 (0-0) | 0 (0-0.01) | 0 (0-0) | 0 (0-0.01) | 0.81 (0.7-0.91) |
| Palestine | 1 (1-2) | 0.03 (0.02-0.06) | 3 (2-4) | 0.06 (0.04-0.08) | 2.18 (1.83-2.52) |
| Panama | 2 (2-2) | 0.08 (0.06-0.09) | 3 (3-4) | 0.08 (0.06-0.1) | -0.13 (-0.26-0) |
| Papua New Guinea | 0 (0-0) | 0 (0-0.01) | 0 (0-1) | 0 (0-0.01) | 0.55 (0.25-0.86) |
| Paraguay | 2 (1-3) | 0.04 (0.03-0.06) | 4 (3-6) | 0.06 (0.04-0.09) | 1.02 (0.8-1.24) |
| Peru | 16 (11-21) | 0.07 (0.05-0.09) | 21 (15-30) | 0.06 (0.04-0.09) | -0.18 (-0.32--0.05) |
| Philippines | 28 (21-37) | 0.04 (0.03-0.05) | 63 (48-75) | 0.06 (0.05-0.07) | 1.14 (1.03-1.26) |
| Poland | 21 (19-23) | 0.06 (0.05-0.06) | 33 (30-37) | 0.07 (0.06-0.08) | 0.66 (0-1.34) |
| Portugal | 12 (10-14) | 0.15 (0.13-0.17) | 10 (8-12) | 0.09 (0.07-0.12) | -1.39 (-1.57--1.21) |
| Puerto Rico | 2 (2-2) | 0.06 (0.05-0.07) | 3 (2-3) | 0.07 (0.06-0.09) | 1.27 (0.99-1.55) |
| Qatar | 0 (0-0) | 0.01 (0-0.01) | 0 (0-0) | 0.01 (0.01-0.02) | 2.32 (1.47-3.18) |
| Republic of Korea | 38 (28-51) | 0.1 (0.07-0.13) | 32 (24-41) | 0.07 (0.05-0.09) | -1.8 (-2.09--1.51) |
| Republic of Moldova | 1 (1-2) | 0.03 (0.02-0.04) | 3 (2-3) | 0.06 (0.05-0.07) | 3.29 (2.95-3.63) |
| Romania | 18 (12-26) | 0.08 (0.05-0.13) | 17 (11-24) | 0.07 (0.05-0.11) | -0.03 (-0.23-0.18) |
| Russian Federation | 125 (84-172) | 0.08 (0.06-0.11) | 128 (112-142) | 0.07 (0.06-0.08) | -1.64 (-2.12--1.17) |
| Rwanda | 6 (3-9) | 0.05 (0.03-0.08) | 8 (4-14) | 0.05 (0.03-0.09) | -0.19 (-0.71-0.32) |
| Saint Kitts and Nevis | 0 (0-0) | 0.04 (0.03-0.06) | 0 (0-0) | 0.09 (0.06-0.13) | 2 (1.73-2.27) |
| Saint Lucia | 0 (0-0) | 0.05 (0.04-0.07) | 0 (0-0) | 0.08 (0.06-0.12) | 1.19 (0.96-1.43) |
| Saint Vincent and the Grenadines | 0 (0-0) | 0 (0-0) | 0 (0-0) | 0.08 (0.06-0.11) | 7.58 (5.1-10.11) |
| Samoa | 0 (0-0) | 0.04 (0.02-0.07) | 0 (0-0) | 0.05 (0.03-0.11) | 1.09 (1.03-1.14) |
| San Marino | 0 (0-0) | 0.03 (0.02-0.04) | 0 (0-0) | 0.02 (0.01-0.03) | -0.73 (-0.98--0.47) |
| Sao Tome and Principe | 0 (0-0) | 0.01 (0-0.02) | 0 (0-0) | 0.01 (0-0.01) | -2.39 (-3.03--1.75) |
| Saudi Arabia | 6 (1-10) | 0.03 (0.01-0.05) | 13 (5-18) | 0.04 (0.02-0.06) | 0.41 (-0.49-1.32) |
| Senegal | 1 (0-2) | 0.01 (0-0.01) | 1 (0-1) | 0.01 (0-0.01) | -3.05 (-3.56--2.54) |
| Serbia | 11 (7-15) | 0.12 (0.08-0.18) | 11 (8-15) | 0.09 (0.07-0.13) | -0.43 (-0.66--0.21) |
| Seychelles | 0 (0-0) | 0 (0-0) | 0 (0-0) | 0 (0-0) | 1.89 (1.62-2.17) |
| Sierra Leone | 0 (0-1) | 0.01 (0-0.01) | 0 (0-0) | 0 (0-0.01) | -3.55 (-4.16--2.94) |
| Singapore | 3 (2-3) | 0.11 (0.09-0.12) | 4 (3-5) | 0.08 (0.07-0.1) | -0.9 (-1.27--0.53) |
| Slovakia | 3 (2-4) | 0.06 (0.05-0.09) | 5 (4-7) | 0.08 (0.05-0.11) | 1.08 (0.87-1.28) |
| Slovenia | 1 (1-1) | 0.06 (0.05-0.07) | 1 (1-2) | 0.04 (0.03-0.06) | -0.18 (-0.72-0.36) |
| Solomon Islands | 0 (0-0) | 0 (0-0.01) | 0 (0-0) | 0 (0-0.01) | 1.09 (0.67-1.52) |
| Somalia | 2 (1-4) | 0.01 (0.01-0.03) | 3 (1-8) | 0.01 (0-0.03) | -1.04 (-1.66--0.42) |
| South Africa | 16 (12-20) | 0.05 (0.03-0.06) | 32 (26-37) | 0.06 (0.05-0.07) | 1.02 (0.79-1.25) |
| South Sudan | 3 (1-6) | 0.03 (0.01-0.06) | 5 (3-8) | 0.03 (0.02-0.06) | 0.75 (0.07-1.44) |
| Spain | 41 (37-46) | 0.12 (0.11-0.14) | 48 (41-57) | 0.1 (0.08-0.12) | -0.29 (-0.45--0.12) |
| Sri Lanka | 8 (6-10) | 0.05 (0.04-0.07) | 16 (10-21) | 0.07 (0.04-0.09) | 1.28 (1.07-1.49) |
| Sudan | 1 (0-3) | 0 (0-0.01) | 3 (1-9) | 0.01 (0-0.02) | 3.79 (3.41-4.17) |
| Suriname | 0 (0-0) | 0.03 (0.02-0.04) | 0 (0-0) | 0.04 (0.03-0.06) | 1.8 (1.57-2.02) |
| Sweden | 7 (6-8) | 0.09 (0.07-0.11) | 10 (8-13) | 0.08 (0.06-0.1) | -0.2 (-0.82-0.42) |
| Switzerland | 6 (5-7) | 0.09 (0.08-0.11) | 14 (11-16) | 0.14 (0.11-0.17) | 0.69 (0.37-1.02) |
| Syrian Arab Republic | 1 (1-3) | 0.01 (0-0.02) | 2 (1-2) | 0.01 (0.01-0.02) | 1.08 (0.83-1.33) |
| Taiwan (Province of China) | 8 (7-9) | 0.05 (0.04-0.05) | 23 (19-27) | 0.08 (0.06-0.09) | 1.74 (1.44-2.05) |
| Tajikistan | 0 (0-0) | 0 (0-0) | 0 (0-0) | 0 (0-0) | 0.06 (-0.36-0.47) |
| Thailand | 24 (18-31) | 0.05 (0.04-0.06) | 50 (38-66) | 0.07 (0.06-0.09) | 1.03 (0.79-1.26) |
| Timor-Leste | 0 (0-0) | 0.01 (0.01-0.02) | 0 (0-0) | 0.02 (0.01-0.03) | 1.02 (0.76-1.29) |
| Togo | 0 (0-1) | 0.01 (0-0.01) | 0 (0-0) | 0 (0-0.01) | -3.34 (-3.84--2.84) |
| Tokelau | 0 (0-0) | 0.01 (0-0.02) | 0 (0-0) | 0.03 (0.01-0.05) | 1.66 (1.2-2.11) |
| Tonga | 0 (0-0) | 0.01 (0-0.01) | 0 (0-0) | 0.01 (0-0.01) | 1.04 (0.74-1.33) |
| Trinidad and Tobago | 2 (2-2) | 0.15 (0.13-0.18) | 3 (2-4) | 0.24 (0.18-0.31) | 1.72 (1.5-1.95) |
| Tunisia | 7 (4-10) | 0.07 (0.05-0.1) | 9 (6-13) | 0.08 (0.05-0.12) | 0.26 (0.13-0.39) |
| Turkey | 48 (30-74) | 0.08 (0.05-0.12) | 91 (68-115) | 0.12 (0.09-0.16) | 1.44 (1.28-1.59) |
| Turkmenistan | 1 (1-1) | 0.03 (0.02-0.04) | 3 (2-4) | 0.06 (0.04-0.09) | 2.55 (2.16-2.95) |
| Tuvalu | 0 (0-0) | 0.01 (0-0.01) | 0 (0-0) | 0.01 (0.01-0.01) | 0.56 (0.36-0.76) |
| Uganda | 11 (6-18) | 0.03 (0.02-0.05) | 39 (19-75) | 0.06 (0.03-0.11) | 1.88 (1.09-2.69) |
| Ukraine | 43 (30-58) | 0.08 (0.06-0.11) | 49 (34-65) | 0.1 (0.07-0.14) | 0.93 (0.71-1.15) |
| United Arab Emirates | 0 (0-1) | 0.03 (0.02-0.05) | 2 (2-3) | 0.04 (0.03-0.06) | 1.24 (0.35-2.13) |
| United Kingdom | 82 (80-85) | 0.16 (0.15-0.16) | 71 (67-75) | 0.1 (0.09-0.1) | -1.07 (-1.34--0.8) |
| United Republic of Tanzania | 21 (13-34) | 0.05 (0.03-0.08) | 54 (26-94) | 0.07 (0.04-0.12) | 1.5 (0.84-2.17) |
| United States of America | 251 (231-267) | 0.11 (0.1-0.11) | 311 (282-336) | 0.09 (0.08-0.1) | -0.24 (-0.44--0.04) |
| United States Virgin Islands | 0 (0-0) | 0.04 (0.03-0.06) | 0 (0-0) | 0.07 (0.04-0.11) | 2.09 (1.82-2.37) |
| Uruguay | 3 (2-4) | 0.09 (0.07-0.11) | 4 (3-5) | 0.11 (0.08-0.13) | 0.84 (0.7-0.98) |
| Uzbekistan | 6 (3-10) | 0.04 (0.02-0.06) | 21 (14-30) | 0.07 (0.05-0.1) | 2.65 (2.27-3.04) |
| Vanuatu | 0 (0-0) | 0 (0-0.01) | 0 (0-0) | 0.01 (0-0.01) | 0.87 (0.48-1.26) |
| Venezuela (Bolivarian Republic of) | 16 (13-19) | 0.08 (0.07-0.1) | 25 (18-33) | 0.09 (0.07-0.12) | 0.54 (0.29-0.8) |
| Viet Nam | 26 (17-36) | 0.03 (0.02-0.05) | 48 (34-66) | 0.05 (0.04-0.07) | 0.73 (0.54-0.92) |
| Yemen | 0 (0-1) | 0 (0-0.01) | 1 (1-4) | 0 (0-0.01) | 4.69 (4.27-5.11) |
| Zambia | 8 (5-13) | 0.06 (0.04-0.09) | 21 (11-37) | 0.09 (0.05-0.15) | 1.25 (0.57-1.92) |
| Zimbabwe | 3 (2-4) | 0.02 (0.02-0.03) | 7 (5-11) | 0.04 (0.03-0.06) | 1.94 (1.69-2.19) |
