# Supplementary material for: Global burden, trends, and projections to 2050 of neuroblastoma and other peripheral nervous cell tumors: a systematic analysis of the global burden of disease study from 1990 to 2021
Source: Front Pediatr. 2025 Sep 3;13:1604053. doi: 10.3389/fped.2025.1604053 (PMC12440315; doi:10.3389/fped.2025.1604053)
Supplement: Supplementary file 2 [file Table2.docx]

**Table S2. The number of DALYs cases and age-standardized rates of Neuroblastoma and other peripheral neuroblastoma DALYs across countries and territories in 1990 and 2021, and its trends from 1990 to 2021 globally.**

| Characteristics | 1990 | | 2021 | | 1990–2021 |
| --- | --- | --- | --- | --- | --- |
|  | Number of DALYs  cases(95% UI) | The age-standardized DALYs  rate/100000(95% UI) | Number of DALYs  cases(95% UI) | The age-standardized DALYs  rate/100000(95% UI) | EAPC(95% CI) |
| Afghanistan | 12 (2-65) | 0.09 (0.02-0.49) | 96 (38-331) | 0.25 (0.1-0.85) | 3.78 (3.28-4.29) |
| Albania | 50 (34-70) | 1.31 (0.9-1.82) | 33 (18-55) | 1.82 (0.89-3.13) | -0.02 (-0.43-0.4) |
| Algeria | 1225 (731-1956) | 3.48 (2.11-5.46) | 1307 (893-1996) | 2.87 (1.97-4.35) | -1.08 (-1.26--0.89) |
| American Samoa | 0 (0-1) | 1.05 (0.52-1.69) | 1 (0-1) | 1.04 (0.59-1.99) | -0.48 (-1.1-0.15) |
| Andorra | 3 (2-5) | 8.55 (5.33-13.7) | 3 (2-4) | 4.36 (2.77-6.44) | -1.32 (-1.87--0.76) |
| Angola | 151 (58-365) | 0.95 (0.47-2.08) | 499 (309-817) | 1.18 (0.72-1.87) | 0.61 (0.07-1.17) |
| Antigua and Barbuda | 2 (1-3) | 3.47 (2.01-5.24) | 3 (2-4) | 4.8 (3.34-6.96) | 0.93 (0.6-1.26) |
| Argentina | 1699 (1351-2182) | 5.01 (3.99-6.4) | 2225 (1745-2810) | 5.71 (4.38-7.43) | 0.57 (0.3-0.84) |
| Armenia | 39 (23-68) | 1.17 (0.69-2.02) | 123 (80-185) | 3.68 (2.31-5.63) | 5.1 (4.64-5.56) |
| Australia | 891 (784-1013) | 6.17 (5.38-7.12) | 1194 (965-1458) | 5.67 (4.5-7.18) | -0.5 (-0.8--0.19) |
| Austria | 393 (346-451) | 6.76 (5.92-7.83) | 274 (212-351) | 3.97 (2.91-5.24) | -1.49 (-1.9--1.07) |
| Azerbaijan | 97 (60-150) | 1.34 (0.83-2.05) | 140 (86-216) | 1.41 (0.86-2.23) | 0.7 (0.4-1.01) |
| Bahamas | 14 (11-19) | 5.3 (4.03-7.03) | 20 (14-28) | 6.63 (4.6-9.9) | 0.38 (0.11-0.66) |
| Bahrain | 5 (3-10) | 1.06 (0.65-1.83) | 32 (20-46) | 2.74 (1.66-3.93) | 4.17 (3.66-4.69) |
| Bangladesh | 3155 (1779-5123) | 1.81 (1.07-2.98) | 3834 (2209-5920) | 2.51 (1.43-3.88) | 0.09 (-0.31-0.5) |
| Barbados | 28 (22-35) | 12.67 (9.79-16.16) | 28 (21-39) | 13.03 (9.12-18.73) | 0.11 (-0.2-0.42) |
| Belarus | 439 (323-581) | 4.91 (3.55-6.58) | 534 (391-706) | 5.18 (3.8-6.96) | 0.07 (-0.24-0.38) |
| Belgium | 483 (393-588) | 6.49 (5.23-7.96) | 464 (366-579) | 5.15 (3.89-6.67) | -0.93 (-1.28--0.58) |
| Belize | 16 (12-21) | 5.83 (4.49-7.51) | 29 (22-38) | 7.12 (5.44-9.54) | 0.31 (-0.33-0.96) |
| Benin | 43 (21-77) | 0.51 (0.26-0.88) | 28 (15-53) | 0.19 (0.11-0.32) | -4.68 (-5.3--4.05) |
| Bermuda | 1 (1-1) | 1.9 (1.4-2.66) | 1 (1-2) | 3.06 (2.23-4.18) | 1.59 (0.92-2.26) |
| Bhutan | 13 (5-27) | 1.37 (0.54-2.99) | 16 (7-30) | 2.49 (1.07-4.55) | 1.2 (0.89-1.5) |
| Bolivia (Plurinational State of) | 373 (227-590) | 4.27 (2.73-6.62) | 560 (376-795) | 4.79 (3.23-6.76) | -0.28 (-0.51--0.04) |
| Bosnia and Herzegovina | 73 (47-118) | 1.78 (1.12-2.91) | 72 (50-100) | 2.32 (1.57-3.31) | 1.62 (1.31-1.93) |
| Botswana | 21 (13-33) | 1.31 (0.79-2.05) | 68 (44-114) | 3.03 (1.99-4.95) | 2.69 (2.51-2.88) |
| Brazil | 9680 (8448-10964) | 5.93 (5.19-6.7) | 12199 (10343-14121) | 6.41 (5.33-7.55) | 0.02 (-0.32-0.35) |
| Brunei Darussalam | 16 (11-26) | 5.43 (3.58-8.34) | 18 (13-25) | 4.92 (3.52-6.79) | -0.52 (-0.79--0.25) |
| Bulgaria | 181 (111-277) | 2.68 (1.6-4.2) | 180 (115-271) | 3.05 (1.81-4.86) | -0.35 (-0.91-0.22) |
| Burkina Faso | 51 (22-101) | 0.32 (0.14-0.62) | 32 (16-67) | 0.13 (0.07-0.23) | -4.1 (-4.72--3.48) |
| Burundi | 257 (148-469) | 2.56 (1.47-4.64) | 341 (115-828) | 1.75 (0.62-4.16) | -0.69 (-1.43-0.05) |
| Cabo Verde | 0 (0-0) | 0.02 (0.01-0.03) | 0 (0-1) | 0.07 (0.04-0.1) | 4.87 (4.58-5.16) |
| Cambodia | 131 (70-210) | 0.88 (0.5-1.44) | 217 (133-362) | 1.29 (0.78-2.14) | 0.53 (0.26-0.81) |
| Cameroon | 108 (51-191) | 0.65 (0.33-1.1) | 92 (59-174) | 0.29 (0.19-0.5) | -3.88 (-4.4--3.35) |
| Canada | 1592 (1368-1864) | 7.2 (6.08-8.55) | 1575 (1293-2052) | 5.47 (4.24-7.6) | -0.96 (-1.32--0.6) |
| Central African Republic | 28 (14-52) | 0.7 (0.41-1.21) | 46 (25-81) | 0.69 (0.39-1.17) | -0.19 (-0.59-0.22) |
| Chad | 25 (12-49) | 0.25 (0.12-0.47) | 22 (10-52) | 0.12 (0.06-0.21) | -3.78 (-4.4--3.16) |
| Chile | 205 (169-248) | 1.5 (1.25-1.82) | 626 (495-789) | 3.86 (2.99-4.97) | 2.85 (2.09-3.62) |
| China | 22017 (16940-28512) | 1.99 (1.53-2.58) | 42337 (31944-50792) | 3.31 (2.49-4) | 1.91 (1.54-2.3) |
| Colombia | 1403 (1128-1713) | 3.7 (2.99-4.46) | 2059 (1538-2680) | 4.8 (3.53-6.34) | 0.52 (0.02-1.03) |
| Comoros | 26 (13-47) | 3.33 (1.63-5.88) | 45 (23-79) | 5.56 (2.87-9.82) | 0.73 (0.23-1.23) |
| Congo | 50 (26-89) | 1.61 (0.95-2.63) | 99 (64-151) | 1.79 (1.18-2.64) | 0.05 (-0.22-0.32) |
| Cook Islands | 0 (0-0) | 0.39 (0.18-0.67) | 0 (0-0) | 0.63 (0.32-1.1) | 1.37 (1.26-1.49) |
| Costa Rica | 168 (130-212) | 4.59 (3.59-5.73) | 193 (151-239) | 4.47 (3.46-5.74) | -0.68 (-0.85--0.5) |
| Côte d'Ivoire | 186 (47-349) | 0.87 (0.27-1.6) | 421 (126-860) | 1.05 (0.36-2.07) | 0.07 (-0.58-0.73) |
| Croatia | 240 (191-289) | 5.88 (4.61-7.18) | 298 (220-391) | 7.62 (5.51-10.26) | 0.73 (0.44-1.02) |
| Cuba | 510 (404-659) | 5.29 (4.16-6.83) | 455 (353-575) | 5.32 (3.98-7.02) | 0.63 (0.13-1.13) |
| Cyprus | 35 (24-53) | 4.87 (3.31-7.5) | 56 (41-73) | 4.96 (3.55-6.91) | 0.27 (-0.12-0.67) |
| Czechia | 413 (326-534) | 4.7 (3.69-6.17) | 481 (343-672) | 4.58 (3.19-6.55) | -0.2 (-0.52-0.12) |
| Democratic People's Republic of Korea | 436 (278-686) | 1.98 (1.28-3.11) | 365 (228-568) | 1.59 (0.92-2.62) | -0.74 (-0.93--0.54) |
| Democratic Republic of the Congo | 461 (225-835) | 0.8 (0.49-1.3) | 627 (361-1051) | 0.61 (0.36-1.04) | -1.01 (-1.59--0.42) |
| Denmark | 132 (109-160) | 3.17 (2.52-3.94) | 295 (230-376) | 6.14 (4.49-8.31) | 1.43 (1.06-1.81) |
| Djibouti | 30 (14-52) | 4.78 (2.21-8.28) | 70 (31-130) | 5.02 (2.27-9.19) | -0.12 (-0.56-0.32) |
| Dominica | 3 (2-4) | 3.13 (1.95-4.79) | 5 (3-8) | 10.54 (5.69-18.22) | 3.11 (2.76-3.46) |
| Dominican Republic | 204 (122-457) | 2.16 (1.3-4.78) | 673 (430-981) | 6.43 (4.1-9.45) | 1.83 (1.26-2.4) |
| Ecuador | 305 (235-399) | 2.65 (2.07-3.42) | 765 (585-1048) | 4.36 (3.33-5.96) | 1.84 (1.43-2.26) |
| Egypt | 936 (442-2102) | 1.27 (0.65-2.74) | 1532 (959-3013) | 1.33 (0.84-2.6) | 0.05 (-0.4-0.49) |
| El Salvador | 121 (88-164) | 1.8 (1.33-2.37) | 120 (87-164) | 1.91 (1.39-2.6) | -0.37 (-0.6--0.15) |
| Equatorial Guinea | 5 (3-9) | 0.79 (0.48-1.37) | 35 (20-61) | 2.29 (1.33-3.69) | 2.97 (2.52-3.41) |
| Eritrea | 131 (75-217) | 2.28 (1.34-3.79) | 366 (174-677) | 4.26 (2.08-7.75) | 1.46 (0.87-2.04) |
| Estonia | 68 (47-99) | 4.92 (3.42-7.21) | 83 (55-118) | 5.43 (3.62-8.08) | 0.29 (0.02-0.55) |
| Eswatini | 15 (10-25) | 1.41 (0.91-2.16) | 30 (18-49) | 2.59 (1.65-4.11) | 1.69 (1.49-1.88) |
| Ethiopia | 1721 (754-5511) | 1.97 (0.95-5.92) | 4342 (1872-11587) | 2.98 (1.28-7.58) | 0.99 (0.3-1.68) |
| Fiji | 12 (7-19) | 1.61 (1.02-2.47) | 13 (7-31) | 1.46 (0.77-3.39) | -1.81 (-2.38--1.23) |
| Finland | 124 (104-149) | 2.78 (2.31-3.41) | 345 (278-436) | 7.98 (6.17-10.56) | 3.01 (2.51-3.5) |
| France | 2959 (2635-3306) | 6.35 (5.6-7.11) | 2717 (2189-3396) | 5.06 (3.97-6.48) | -0.72 (-0.99--0.46) |
| Gabon | 18 (11-34) | 1.5 (0.95-2.64) | 43 (27-63) | 2.31 (1.47-3.29) | 1.2 (0.98-1.42) |
| Gambia | 27 (8-50) | 1.61 (0.58-2.89) | 59 (23-120) | 1.84 (0.82-3.6) | -0.38 (-1.02-0.27) |
| Georgia | 15 (10-23) | 0.27 (0.17-0.42) | 189 (127-270) | 4.66 (3.1-6.74) | 13.35 (12.41-14.29) |
| Germany | 3556 (2939-4255) | 5.74 (4.71-6.95) | 4067 (3256-5057) | 6.31 (4.83-8.21) | -0.05 (-0.5-0.4) |
| Ghana | 20 (11-32) | 0.16 (0.07-0.27) | 34 (21-51) | 0.12 (0.07-0.18) | -2.77 (-3.56--1.97) |
| Greece | 215 (194-239) | 2.74 (2.44-3.06) | 208 (182-239) | 2.67 (2.24-3.16) | 0.33 (-0.11-0.78) |
| Greenland | 3 (2-4) | 5.11 (3.57-7.13) | 1 (1-1) | 1.63 (1.06-2.37) | -3.48 (-3.67--3.28) |
| Grenada | 3 (2-4) | 2.82 (1.98-4.1) | 4 (3-5) | 4.74 (3.48-6.47) | 1.44 (1.15-1.74) |
| Guam | 0 (0-1) | 0.33 (0.24-0.41) | 1 (1-1) | 0.54 (0.42-0.69) | 2.66 (1.91-3.42) |
| Guatemala | 188 (122-298) | 1.48 (1.02-2.26) | 194 (147-258) | 1.24 (0.94-1.66) | -1.39 (-1.77--1) |
| Guinea | 264 (74-461) | 2.4 (0.74-4.19) | 488 (137-1043) | 2.3 (0.75-4.8) | -0.19 (-0.93-0.55) |
| Guinea-Bissau | 8 (4-14) | 0.54 (0.3-0.86) | 4 (2-7) | 0.21 (0.13-0.33) | -4.51 (-4.96--4.06) |
| Guyana | 3 (2-5) | 0.32 (0.23-0.45) | 45 (30-67) | 6.06 (3.94-9) | 7.74 (6.05-9.46) |
| Haiti | 363 (121-717) | 3.73 (1.38-7.17) | 770 (367-1363) | 5.12 (2.52-8.92) | 1.02 (0.63-1.41) |
| Honduras | 154 (102-235) | 2.26 (1.51-3.48) | 288 (174-438) | 2.88 (1.78-4.28) | 0.02 (-0.27-0.32) |
| Hungary | 455 (380-541) | 5.27 (4.35-6.34) | 496 (371-669) | 5.3 (3.85-7.29) | 0.02 (-0.44-0.48) |
| Iceland | 12 (10-14) | 5.04 (4.16-6.06) | 18 (14-24) | 6.07 (4.44-8.4) | 1.11 (0.77-1.45) |
| India | 23100 (13981-32002) | 2.12 (1.28-2.94) | 34612 (25595-44339) | 2.85 (2.08-3.7) | 0.18 (-0.1-0.46) |
| Indonesia | 3074 (1917-4925) | 1.47 (0.95-2.29) | 6024 (4509-8129) | 2.42 (1.8-3.34) | 1.11 (1-1.22) |
| Iran (Islamic Republic of) | 183 (101-319) | 0.26 (0.14-0.44) | 547 (183-766) | 0.69 (0.24-0.98) | 4.33 (3.86-4.8) |
| Iraq | 410 (258-643) | 1.52 (0.98-2.34) | 1186 (758-1800) | 2.8 (1.84-4.2) | 1.8 (1.42-2.18) |
| Ireland | 216 (181-262) | 6.56 (5.53-7.95) | 170 (134-219) | 4.04 (3.07-5.28) | -1.37 (-1.63--1.11) |
| Israel | 360 (271-454) | 7.01 (5.28-8.85) | 436 (332-566) | 4.61 (3.49-6.01) | -1.29 (-1.53--1.05) |
| Italy | 3571 (3281-3846) | 10.14 (9.26-11.01) | 3159 (2619-3700) | 8.79 (6.85-10.77) | -0.4 (-1.06-0.26) |
| Jamaica | 132 (102-173) | 4.9 (3.81-6.37) | 225 (149-324) | 10.4 (6.74-15.38) | 1.84 (1.25-2.44) |
| Japan | 6215 (5975-6452) | 7.49 (7.17-7.81) | 5861 (5415-6292) | 7.35 (6.61-8.04) | -0.45 (-1.11-0.22) |
| Jordan | 171 (116-257) | 3.31 (2.28-4.94) | 489 (334-726) | 4.2 (2.85-6.3) | 0.25 (-0.08-0.57) |
| Kazakhstan | 127 (94-172) | 0.8 (0.58-1.08) | 255 (179-366) | 1.32 (0.93-1.89) | 1.61 (1.47-1.75) |
| Kenya | 456 (271-815) | 1.16 (0.68-2.04) | 1103 (650-1697) | 1.98 (1.19-2.99) | 1.55 (1.01-2.09) |
| Kiribati | 0 (0-0) | 0.1 (0.06-0.14) | 0 (0-0) | 0.14 (0.08-0.21) | 0.76 (0.62-0.9) |
| Kuwait | 52 (40-69) | 2.86 (2.2-3.73) | 105 (77-141) | 3.23 (2.28-4.48) | 3.17 (1.79-4.56) |
| Kyrgyzstan | 125 (73-204) | 2.1 (1.26-3.37) | 97 (69-135) | 1.48 (1.06-2.04) | 4.37 (2.69-6.07) |
| Lao People's Democratic Republic | 45 (20-85) | 0.77 (0.39-1.35) | 101 (59-166) | 1.34 (0.78-2.18) | 1.5 (1.21-1.79) |
| Latvia | 91 (65-135) | 3.95 (2.76-5.95) | 60 (41-86) | 2.63 (1.79-3.91) | 0.06 (-0.29-0.42) |
| Lebanon | 72 (48-104) | 2.04 (1.39-2.93) | 128 (82-195) | 2.69 (1.65-4.26) | 0.79 (0.62-0.97) |
| Lesotho | 14 (9-24) | 0.75 (0.44-1.31) | 29 (18-43) | 1.61 (1.04-2.4) | 2.57 (2.35-2.78) |
| Liberia | 21 (11-35) | 0.53 (0.28-0.82) | 7 (4-14) | 0.14 (0.07-0.25) | -5.36 (-6.14--4.58) |
| Libya | 215 (123-377) | 3.8 (2.17-6.44) | 379 (228-591) | 7.15 (4.13-11.39) | 1.49 (1.13-1.84) |
| Lithuania | 111 (77-157) | 3.4 (2.33-4.83) | 145 (103-193) | 4.5 (3.18-5.97) | 1.59 (1.35-1.83) |
| Luxembourg | 18 (16-21) | 6.5 (5.61-7.43) | 17 (14-21) | 3.56 (2.85-4.45) | -2.34 (-2.68--1.99) |
| Madagascar | 705 (441-1097) | 3.41 (2.18-5.31) | 1223 (627-2085) | 3.2 (1.7-5.33) | -0.32 (-1.03-0.38) |
| Malawi | 2074 (1357-3108) | 11.07 (7.24-16.65) | 3273 (1336-6636) | 12.55 (5.31-25.11) | 0.23 (-0.54-1) |
| Malaysia | 584 (345-835) | 2.94 (1.78-4.11) | 1382 (1033-1689) | 4.71 (3.51-5.87) | 1.19 (0.95-1.42) |
| Maldives | 17 (7-42) | 5.11 (2.4-11.68) | 33 (24-47) | 8.99 (6.19-12.83) | 0.81 (0.41-1.21) |
| Mali | 159 (36-294) | 0.99 (0.27-1.79) | 375 (100-833) | 0.91 (0.3-1.94) | -0.41 (-1.22-0.4) |
| Malta | 38 (30-47) | 11.91 (9.24-15.14) | 39 (30-52) | 12.52 (8.9-17.47) | -0.16 (-0.56-0.24) |
| Marshall Islands | 0 (0-0) | 0.28 (0.13-0.55) | 0 (0-0) | 0.43 (0.25-0.67) | 1.09 (0.91-1.26) |
| Mauritania | 19 (9-33) | 0.61 (0.32-1.01) | 12 (7-25) | 0.28 (0.17-0.51) | -4.3 (-4.79--3.82) |
| Mauritius | 15 (13-18) | 1.46 (1.28-1.67) | 41 (36-46) | 3.75 (3.08-4.35) | 2.97 (2.33-3.6) |
| Mexico | 4139 (3769-4612) | 3.82 (3.5-4.24) | 5039 (4267-5923) | 4.28 (3.57-5.14) | -0.31 (-0.79-0.17) |
| Micronesia (Federated States of) | 0 (0-1) | 0.36 (0.18-0.62) | 0 (0-1) | 0.39 (0.23-0.63) | 0 (-0.09-0.09) |
| Monaco | 0 (0-0) | 0.1 (0.07-0.16) | 0 (0-0) | 0.14 (0.08-0.23) | -0.04 (-0.51-0.43) |
| Mongolia | 29 (16-50) | 1.26 (0.75-2.03) | 74 (49-103) | 2.27 (1.52-3.14) | 2.13 (1.94-2.31) |
| Montenegro | 20 (14-28) | 3.55 (2.35-5.06) | 15 (11-20) | 2.49 (1.75-3.46) | -0.23 (-0.46-0.01) |
| Morocco | 1322 (846-1943) | 3.94 (2.61-5.71) | 1683 (1035-2658) | 4.94 (3.01-7.9) | 0.5 (0.27-0.73) |
| Mozambique | 733 (416-1370) | 3.13 (1.79-5.86) | 1557 (600-3517) | 3.28 (1.32-7.2) | 0.26 (-0.52-1.04) |
| Myanmar | 569 (276-995) | 1.21 (0.63-2.08) | 1056 (714-1523) | 1.97 (1.33-2.85) | 1.43 (1.33-1.52) |
| Namibia | 51 (27-78) | 2.58 (1.46-3.87) | 142 (79-229) | 5.32 (3.05-8.5) | 2.27 (1.93-2.62) |
| Nauru | 0 (0-0) | 0.62 (0.29-1.08) | 0 (0-0) | 0.69 (0.44-0.99) | 0.13 (-0.04-0.3) |
| Nepal | 395 (227-708) | 1.28 (0.74-2.32) | 504 (242-841) | 1.61 (0.78-2.69) | 0.2 (-0.17-0.57) |
| Netherlands | 1031 (882-1216) | 8.94 (7.56-10.77) | 828 (655-1039) | 5.84 (4.46-7.65) | -1.65 (-2.02--1.28) |
| New Zealand | 214 (181-254) | 6.99 (5.88-8.36) | 230 (193-271) | 4.86 (4-5.85) | -0.61 (-1.05--0.16) |
| Nicaragua | 350 (237-517) | 5.98 (4.22-8.58) | 267 (199-376) | 4.1 (3.07-5.76) | -1.76 (-2.07--1.44) |
| Niger | 44 (20-93) | 0.3 (0.14-0.62) | 17 (6-38) | 0.07 (0.02-0.14) | -6.54 (-7.25--5.83) |
| Nigeria | 6230 (1753-9975) | 4.52 (1.34-7.33) | 24947 (6371-43076) | 7.78 (2.3-12.9) | 2.03 (1.42-2.64) |
| Niue | 0 (0-0) | 0.55 (0.29-0.85) | 0 (0-0) | 1.49 (0.99-2.21) | 1.28 (0.89-1.68) |
| North Macedonia | 60 (45-82) | 3.37 (2.5-4.61) | 45 (32-61) | 2.43 (1.6-3.47) | -0.44 (-0.78--0.1) |
| Northern Mariana Islands | 0 (0-0) | 0.09 (0.05-0.14) | 0 (0-0) | 0.1 (0.08-0.14) | 0.7 (0.21-1.18) |
| Norway | 196 (183-209) | 5.29 (4.91-5.64) | 210 (184-240) | 3.96 (3.44-4.57) | -1.73 (-2.07--1.4) |
| Oman | 42 (12-85) | 1.52 (0.46-3.02) | 86 (51-118) | 2.02 (1.23-2.79) | 0.32 (-0.08-0.73) |
| Pakistan | 7909 (5085-12358) | 4.47 (2.95-6.86) | 26664 (17037-41032) | 9.32 (6.07-14.19) | 2.74 (2.22-3.26) |
| Palau | 0 (0-0) | 0.19 (0.1-0.35) | 0 (0-0) | 0.25 (0.14-0.37) | 0.8 (0.68-0.93) |
| Palestine | 71 (41-127) | 2.24 (1.33-3.83) | 207 (134-299) | 3.61 (2.38-5.08) | 2.02 (1.47-2.57) |
| Panama | 131 (107-160) | 4.92 (4.06-5.96) | 199 (155-256) | 4.99 (3.87-6.48) | -0.57 (-0.73--0.42) |
| Papua New Guinea | 7 (2-19) | 0.16 (0.04-0.48) | 21 (9-45) | 0.2 (0.08-0.45) | 0.47 (0.35-0.58) |
| Paraguay | 171 (104-256) | 3.05 (1.88-4.5) | 284 (179-446) | 4.19 (2.63-6.61) | 0.68 (0.38-0.98) |
| Peru | 1187 (791-1641) | 4.48 (3.04-6.15) | 1092 (770-1500) | 3.13 (2.21-4.29) | -1.25 (-1.41--1.08) |
| Philippines | 2162 (1551-2978) | 2.74 (2.05-3.59) | 3866 (2968-4747) | 3.49 (2.68-4.25) | 0.71 (0.51-0.91) |
| Poland | 1210 (1118-1310) | 3.6 (3.3-3.91) | 1215 (1075-1343) | 3.52 (3.03-4.05) | -0.34 (-1.02-0.35) |
| Portugal | 795 (667-926) | 10.89 (9.04-12.87) | 401 (320-520) | 5.55 (4.25-7.56) | -2.43 (-2.89--1.96) |
| Puerto Rico | 125 (99-156) | 3.68 (2.91-4.64) | 103 (80-127) | 4.21 (3.27-5.5) | 0.45 (0.03-0.87) |
| Qatar | 2 (1-3) | 0.4 (0.27-0.6) | 12 (8-18) | 0.62 (0.38-0.95) | 2.06 (1.41-2.72) |
| Republic of Korea | 2681 (1885-3648) | 6.75 (4.71-9.21) | 1454 (1085-1873) | 4.5 (3.03-6.37) | -2.62 (-3.09--2.14) |
| Republic of Moldova | 74 (58-106) | 1.72 (1.33-2.45) | 94 (76-113) | 2.85 (2.17-3.57) | 2.23 (1.89-2.59) |
| Romania | 1114 (705-1723) | 5.69 (3.54-9.06) | 669 (449-981) | 4.17 (2.72-6.37) | -1.06 (-1.45--0.66) |
| Russian Federation | 6299 (4356-8530) | 4.55 (3.22-6.11) | 4846 (4184-5428) | 3.39 (2.86-3.82) | -2.09 (-2.51--1.68) |
| Rwanda | 492 (297-794) | 3.94 (2.42-6.28) | 657 (298-1174) | 3.97 (1.83-7.03) | -0.32 (-0.93-0.3) |
| Saint Kitts and Nevis | 1 (1-2) | 2.94 (2.09-4.25) | 3 (2-4) | 6.46 (3.95-10.13) | 1.73 (1.36-2.09) |
| Saint Lucia | 6 (4-8) | 3.54 (2.53-4.95) | 7 (5-10) | 5.62 (3.57-8.48) | 0.7 (0.36-1.04) |
| Saint Vincent and the Grenadines | 0 (0-0) | 0.21 (0.16-0.27) | 5 (4-7) | 5.58 (4.04-7.83) | 6.89 (4.39-9.45) |
| Samoa | 4 (2-7) | 2.05 (1.21-3.62) | 7 (3-12) | 2.73 (1.5-4.78) | 0.87 (0.66-1.08) |
| San Marino | 0 (0-0) | 1.94 (1.32-2.84) | 0 (0-0) | 1.01 (0.54-1.82) | -1.47 (-1.97--0.98) |
| Sao Tome and Principe | 1 (1-2) | 0.68 (0.33-1.2) | 1 (0-1) | 0.26 (0.14-0.49) | -4.1 (-4.81--3.39) |
| Saudi Arabia | 501 (96-858) | 2.33 (0.46-3.87) | 713 (271-1058) | 2.03 (0.79-3.12) | -0.75 (-1.66-0.16) |
| Senegal | 73 (33-131) | 0.58 (0.27-1.02) | 35 (18-59) | 0.23 (0.13-0.37) | -4.57 (-5.15--3.99) |
| Serbia | 629 (401-944) | 7.9 (4.86-12.05) | 365 (258-515) | 4.35 (2.81-6.72) | -1.95 (-2.21--1.69) |
| Seychelles | 0 (0-0) | 0.05 (0.01-0.08) | 0 (0-0) | 0.08 (0.01-0.11) | 1.97 (1.69-2.25) |
| Sierra Leone | 35 (17-60) | 0.51 (0.25-0.85) | 15 (8-28) | 0.17 (0.1-0.28) | -4.92 (-5.61--4.23) |
| Singapore | 181 (155-210) | 7.45 (6.26-8.8) | 212 (170-267) | 5.47 (4.21-7.01) | -1.34 (-1.83--0.84) |
| Slovakia | 176 (124-247) | 3.65 (2.53-5.19) | 200 (133-289) | 4.21 (2.72-6.65) | 0.51 (0.2-0.82) |
| Slovenia | 54 (43-66) | 3.28 (2.63-4) | 41 (31-56) | 1.91 (1.34-2.67) | -1.37 (-1.85--0.89) |
| Solomon Islands | 0 (0-1) | 0.15 (0.03-0.44) | 1 (1-3) | 0.22 (0.1-0.45) | 0.99 (0.81-1.17) |
| Somalia | 140 (66-351) | 0.98 (0.46-2.46) | 214 (72-635) | 0.6 (0.21-1.84) | -1.21 (-1.98--0.42) |
| South Africa | 1038 (793-1360) | 2.57 (2.04-3.29) | 1648 (1348-2052) | 3.13 (2.56-3.91) | 0.52 (0.37-0.66) |
| South Sudan | 254 (113-515) | 2.55 (1.14-5.11) | 397 (212-712) | 2.66 (1.45-4.76) | 0.67 (-0.11-1.45) |
| Spain | 2376 (2108-2653) | 8.39 (7.4-9.43) | 1985 (1655-2394) | 6.25 (5.05-7.73) | -0.93 (-1.39--0.47) |
| Sri Lanka | 504 (384-672) | 2.89 (2.22-3.82) | 720 (477-1023) | 3.48 (2.3-5.03) | 0.55 (0.31-0.78) |
| Sudan | 56 (12-241) | 0.18 (0.04-0.75) | 222 (94-705) | 0.44 (0.19-1.36) | 3.45 (2.9-4) |
| Suriname | 8 (5-12) | 1.96 (1.25-2.88) | 16 (10-23) | 3.14 (1.98-4.6) | 1.53 (1.29-1.77) |
| Sweden | 394 (322-480) | 6.08 (4.95-7.48) | 405 (314-527) | 4.81 (3.57-6.42) | -1.05 (-1.64--0.45) |
| Switzerland | 312 (266-365) | 5.78 (4.85-6.83) | 557 (455-681) | 8.28 (6.48-10.79) | 0.21 (-0.27-0.69) |
| Syrian Arab Republic | 107 (59-204) | 0.58 (0.32-1.07) | 90 (55-128) | 0.72 (0.43-1.04) | 0.46 (0.06-0.86) |
| Taiwan (Province of China) | 475 (428-529) | 2.54 (2.29-2.84) | 830 (706-988) | 3.83 (3.19-4.64) | 0.83 (0.5-1.15) |
| Tajikistan | 3 (2-4) | 0.04 (0.03-0.07) | 5 (3-8) | 0.05 (0.03-0.07) | -0.06 (-0.37-0.25) |
| Thailand | 1669 (1195-2208) | 3.06 (2.2-4.02) | 2212 (1717-2800) | 4.25 (3.29-5.46) | 0.13 (-0.2-0.45) |
| Timor-Leste | 9 (4-18) | 0.73 (0.39-1.39) | 16 (10-25) | 1.02 (0.63-1.54) | 0.37 (-0.09-0.82) |
| Togo | 30 (14-57) | 0.52 (0.26-0.93) | 16 (9-28) | 0.2 (0.11-0.32) | -4.78 (-5.34--4.21) |
| Tokelau | 0 (0-0) | 0.44 (0.2-0.71) | 0 (0-0) | 1.74 (0.8-3.59) | 1.75 (1.06-2.45) |
| Tonga | 0 (0-0) | 0.25 (0.11-0.47) | 0 (0-1) | 0.34 (0.18-0.57) | 0.83 (0.68-0.98) |
| Trinidad and Tobago | 139 (113-171) | 10.58 (8.64-13) | 170 (126-221) | 15.74 (11.54-20.96) | 1.23 (0.92-1.54) |
| Tunisia | 534 (354-829) | 5.23 (3.52-8.06) | 525 (335-804) | 5.16 (3.26-8.1) | -0.48 (-0.64--0.32) |
| Turkey | 3781 (2314-5916) | 5.67 (3.47-8.79) | 5159 (3737-7096) | 7.57 (5.24-10.64) | 0.73 (0.52-0.95) |
| Turkmenistan | 48 (33-76) | 1.3 (0.93-1.96) | 117 (75-185) | 2.32 (1.5-3.64) | 2.15 (1.94-2.36) |
| Tuvalu | 0 (0-0) | 0.31 (0.15-0.61) | 0 (0-0) | 0.37 (0.23-0.57) | 0.27 (0.15-0.39) |
| Uganda | 959 (508-1642) | 2.7 (1.47-4.55) | 3411 (1576-6542) | 4.94 (2.39-9.28) | 1.82 (0.93-2.72) |
| Ukraine | 2166 (1578-2940) | 4.7 (3.4-6.57) | 1991 (1396-2690) | 5.79 (3.99-8.27) | 0.63 (0.29-0.96) |
| United Arab Emirates | 31 (19-54) | 1.65 (1.01-2.87) | 125 (89-170) | 2.02 (1.45-2.78) | 0.44 (-0.15-1.03) |
| United Kingdom | 4776 (4614-4966) | 10.9 (10.5-11.35) | 3097 (2873-3303) | 5.86 (5.3-6.36) | -1.64 (-2.1--1.17) |
| United Republic of Tanzania | 1794 (1105-2988) | 3.82 (2.38-6.29) | 4616 (2174-8094) | 5.52 (2.65-9.56) | 1.43 (0.68-2.19) |
| United States of America | 15567 (14417-16617) | 7.31 (6.76-7.81) | 14720 (13123-16173) | 5.54 (4.88-6.16) | -0.77 (-1.08--0.45) |
| United States Virgin Islands | 3 (2-5) | 2.91 (1.97-4.25) | 3 (2-4) | 4.31 (2.51-7.78) | 1.6 (1.22-1.98) |
| Uruguay | 159 (125-205) | 5.38 (4.24-6.98) | 178 (139-224) | 6.02 (4.59-7.83) | 0.21 (-0.01-0.42) |
| Uzbekistan | 332 (210-531) | 1.56 (0.92-2.47) | 1038 (686-1486) | 3.05 (2.01-4.35) | 2.57 (2.38-2.75) |
| Vanuatu | 0 (0-1) | 0.17 (0.05-0.43) | 1 (0-1) | 0.23 (0.12-0.45) | 0.73 (0.58-0.88) |
| Venezuela (Bolivarian Republic of) | 1136 (964-1362) | 5.14 (4.41-6.11) | 1328 (950-1784) | 5.29 (3.76-7.16) | -0.06 (-0.33-0.21) |
| Viet Nam | 2030 (1228-2914) | 2.37 (1.49-3.33) | 2780 (1887-3882) | 3.08 (2.05-4.41) | -0.05 (-0.26-0.16) |
| Yemen | 18 (4-89) | 0.08 (0.02-0.37) | 98 (38-319) | 0.25 (0.1-0.77) | 4.31 (3.65-4.97) |
| Zambia | 703 (407-1172) | 4.8 (2.85-7.92) | 1720 (879-3015) | 6.57 (3.3-11.25) | 0.93 (0.16-1.71) |
| Zimbabwe | 228 (123-359) | 1.52 (0.91-2.3) | 564 (337-852) | 2.91 (1.78-4.29) | 2.04 (1.6-2.48) |
