# Supplementary figures and images for: Global burden, trends, and projections to 2050 of neuroblastoma and other peripheral nervous cell tumors: a systematic analysis of the global burden of disease study from 1990 to 2021
Source: Front Pediatr. 2025 Sep 3;13:1604053. doi: 10.3389/fped.2025.1604053 (PMC12440315; doi:10.3389/fped.2025.1604053)

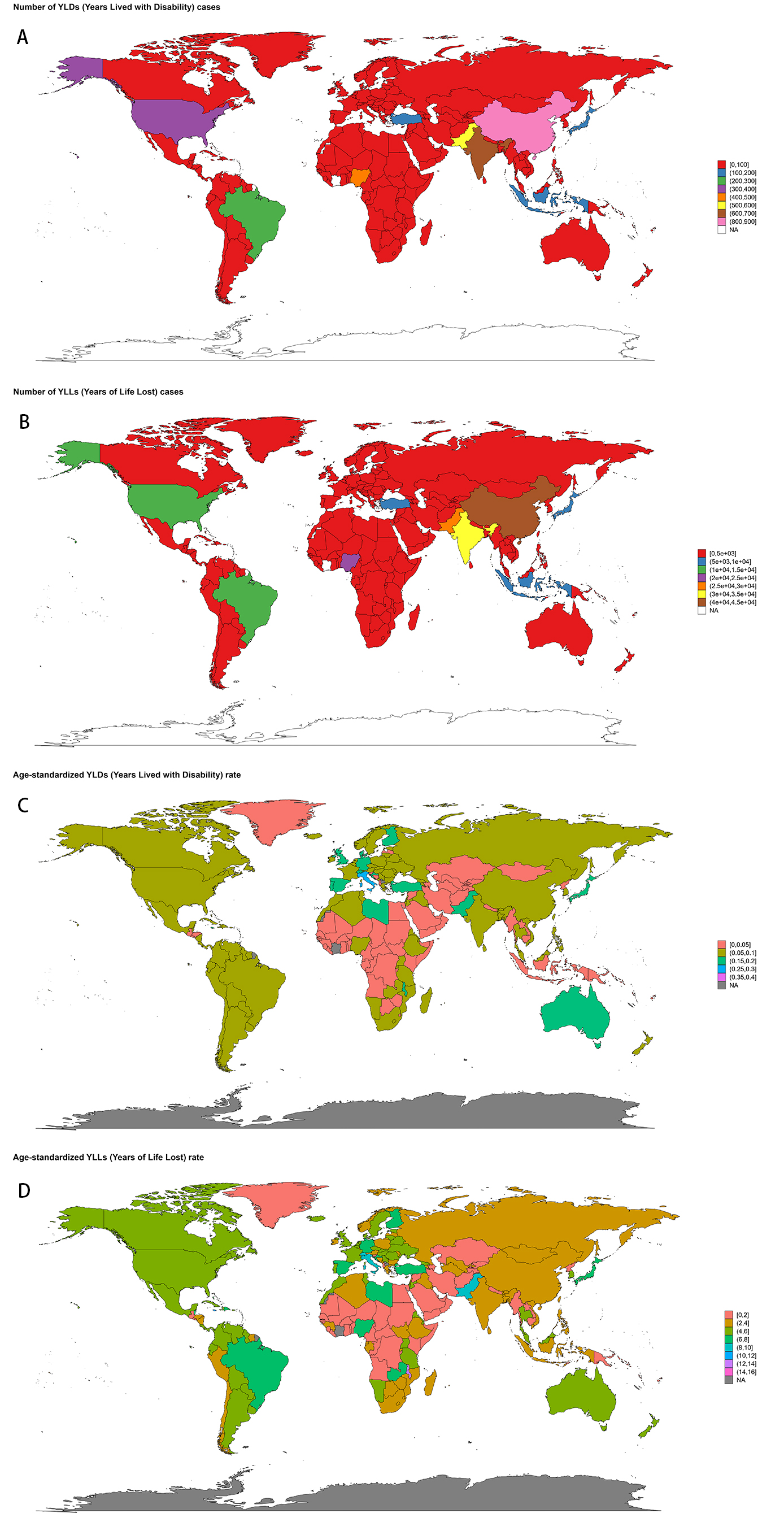

Supplement: Supplementary file 3 [file Image1.tif]

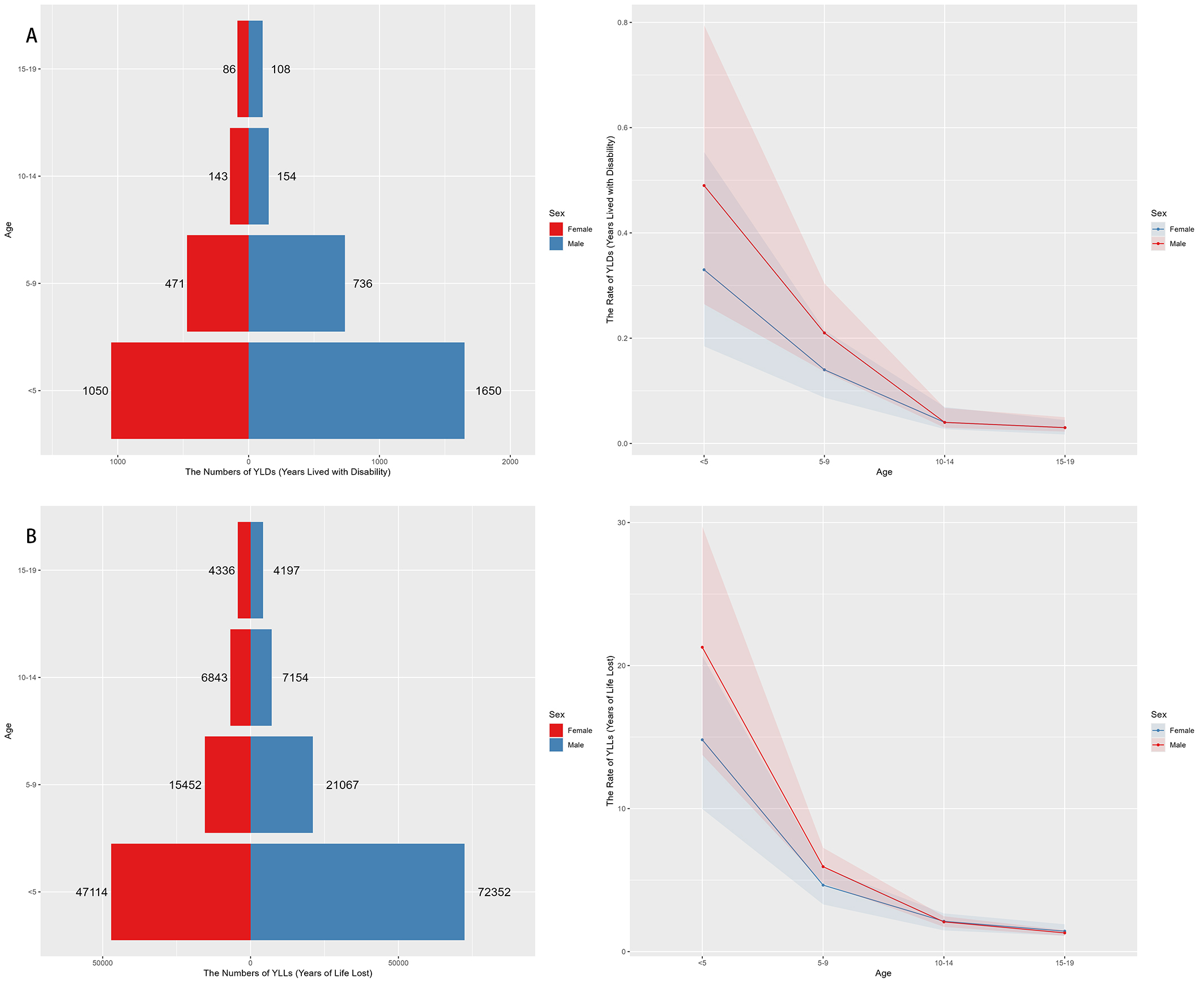

Supplement: Supplementary file 4 [file Image2.tif]

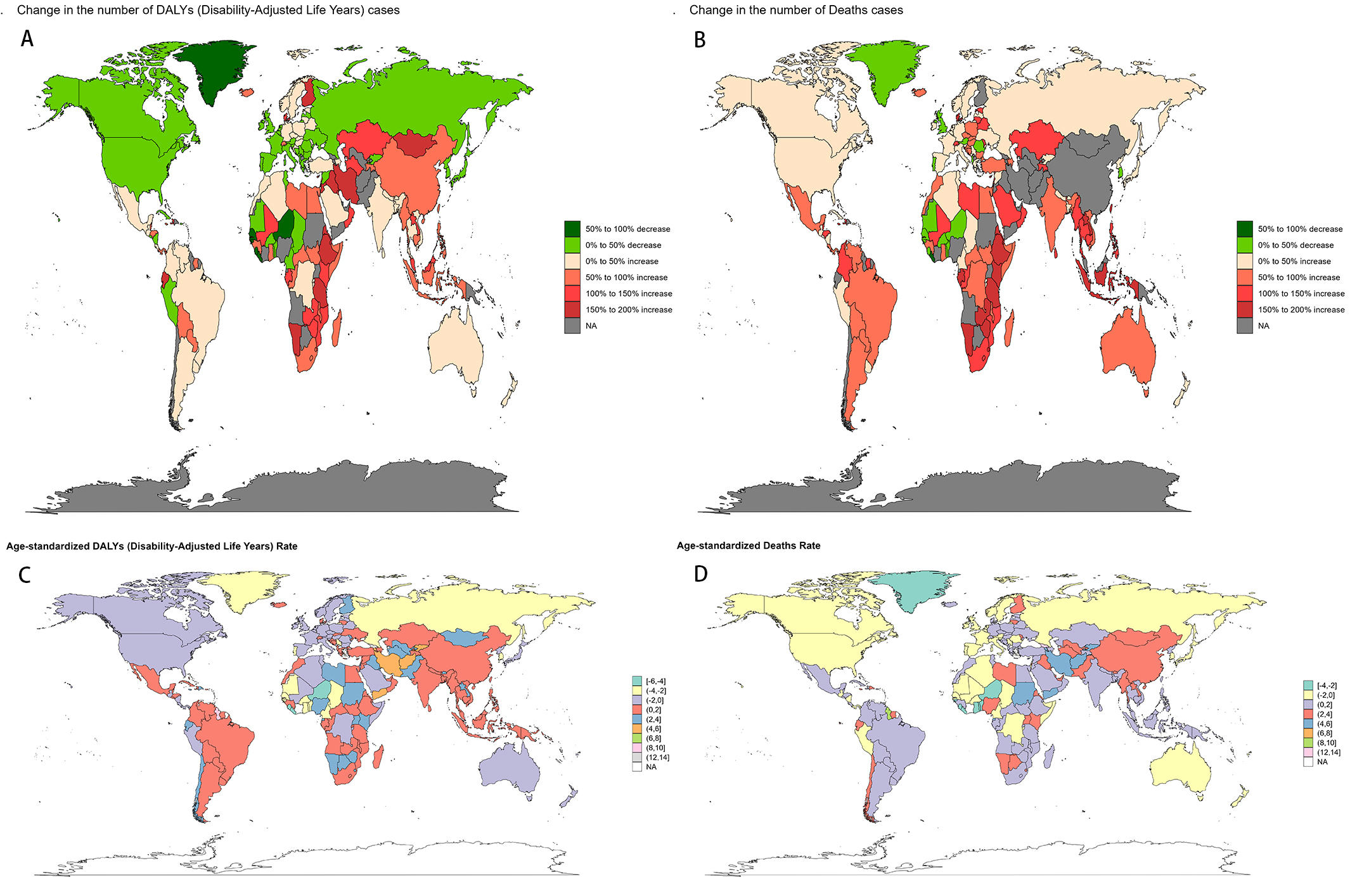

Supplement: Supplementary file 5 [file Image3.tif]

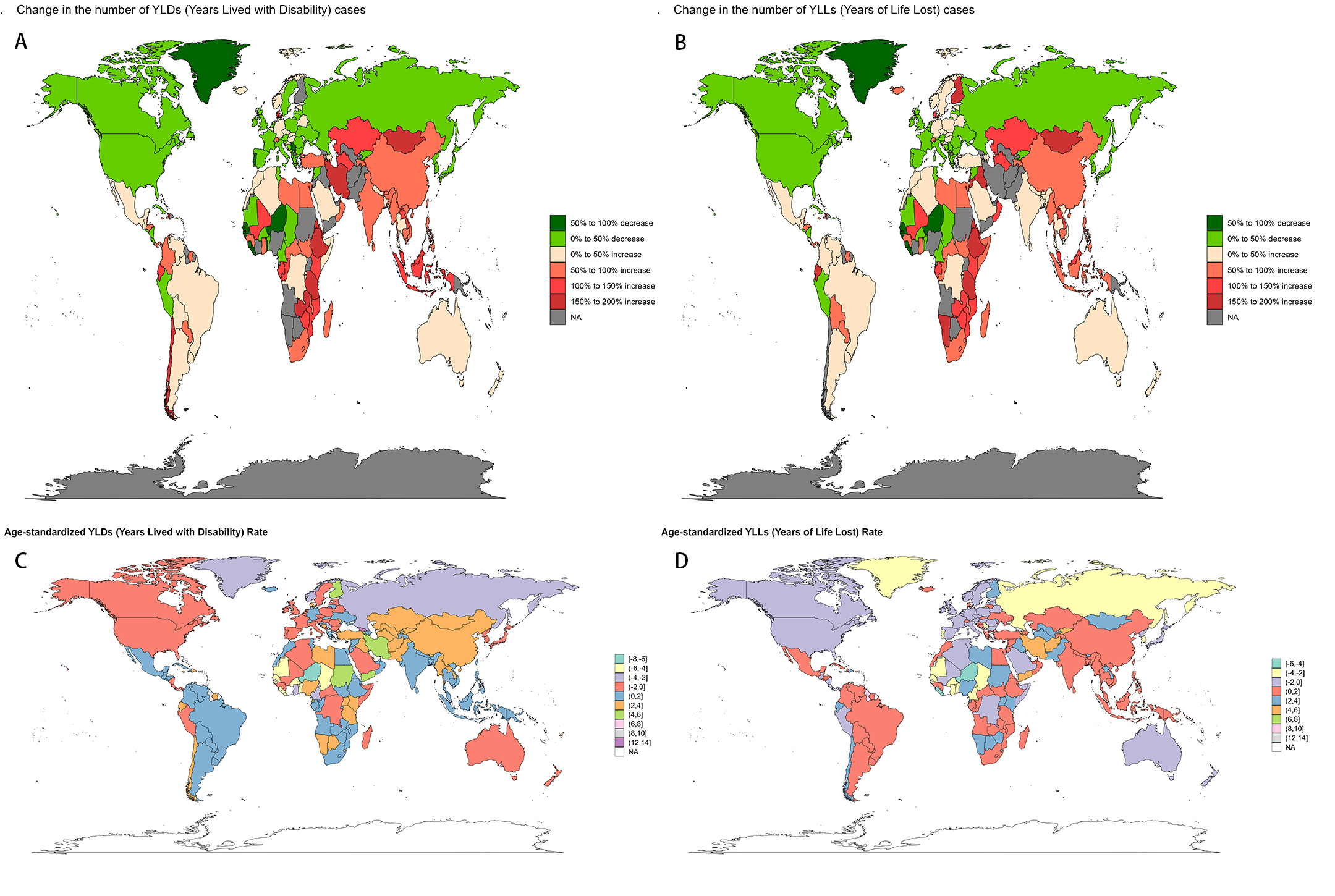

Supplement: Supplementary file 6 [file Image4.tif]
